# i-DENV: development of QSAR based regression models for predicting inhibitors targeting non-structural (NS) proteins of dengue virus

**DOI:** 10.3389/fphar.2025.1605722

**Published:** 2025-06-26

**Authors:** Sakshi Gautam, Anamika Thakur, Manoj Kumar

**Affiliations:** Virology Unit and Bioinformatics Centre, Council of Scientific and Industrial Research (CSIR) - Institute of Microbial Technology, and Academy of Scientific and Innovative Research (AcSIR), Ghaziabad, India

**Keywords:** machine learning, antivirals, artificial intelligence, algorithm, web server, QSAR

## Abstract

**Introduction:**

Dengue virus (DENV) is a significant global arboviral threat with fatal potential, currently lacking effective antiviral treatments or a universally applicable vaccine. In response to this unmet need, we developed the “i‐DENV” web server to facilitate structure‐based drug prediction targeting key viral proteins.

**Methods:**

The i‐DENV platform focuses on the NS3 protease and NS5 polymerase of DENV using machine learning techniques (MLTs) and quantitative structure‐activity relationship (QSAR) modeling. A total of 1213 and 157 unique compounds, along with their IC50 values targeting NS3 and NS5 respectively, were retrieved from the ChEMBL and DenvInD databases. Molecular descriptors and fingerprints were computed and used to train multiple regression‐based MLTs, including SVM, RF, kNN, ANN, XGBoost, and DNN, with ten‐fold cross‐validation.

**Results:**

The best-performing SVM and ANN models achieved Pearson correlation coefficients (PCCs) of 0.857/0.862 (NS3) and 0.982/0.964 (NS5) on training/testing sets, and 0.870/0.894 (NS3) and 0.970/0.977 (NS5) on independent validation sets. Model robustness was supported through scatter plots, chemical clustering, statistical analyses, decoy set etc. Virtual screening identified Micafungin, Oritavancin, and Iodixanol as top hits for NS2B/NS3 protease, and Cangrelor, Eravacycline, and Baloxavir marboxil for NS5 polymerase. Molecular docking further confirmed strong binding affinities of these compounds.

**Discussion:**

Our *in-silico* findings suggest these repurposed drugs as promising antiviral candidates against DENV. However, further *in vitro* and *in vivo* studies are essential to validate their therapeutic potential. The i-DENV web server is freely accessible at http://bioinfo.imtech.res.in/manojk/idenv/, offering a structure-specific drug prediction platform for DENV research and antiviral drug discovery.

## Introduction

Dengue virus (DENV), an arbovirus from the Flaviviridae family, causes tropical diseases and is a major global health concern. The World Health Organization (WHO) lists Dengue among the top ten global threats, with nearly half the world’s population at risk and about 390 million infections annually (Waggoner et al., 2016; Bhatt et al., 2013; Brady et al., 2012). Globalization, urbanization, and climate change are expanding the range of *Aedes aegypti* and *Aedes albopictus*, potentially placing 60% of the global population at risk by 2080 (Ebi and Nealon, 2016; Messina et al., 2019; de Almeida et al., 2017). DENV encompasses four serotypes (DENV-1 to DENV-4), each capable of causing the full range of disease. Infection with a different serotype can lead to severe conditions like dengue hemorrhagic fever (DHF) and potentially fatal dengue shock syndrome (DSS) due to antibody-dependent enhancement (ADE), which complicates vaccine development (Goethals et al., 2023; Dash et al., 2006). Currently, no approved antivirals exist and only symptomatic treatment is available (Kaptein et al., 2021).

DENV possesses an 11 kb single-strand RNA genome, yielding seven non-structural proteins (NS1, NS2A, NS2B, NS3, NS4A, NS4B, NS5) and three structural proteins (CP, EP, MP) (Khan et al., 2023). Its life cycle reveals key steps such as endocytosis, viral fusion, transcription, and release of new viral particles (Behnam et al., 2016). The envelope (E) protein mediates host cell attachment and membrane fusion. The membrane (M) protein stabilizes the mature virion and aids assembly after prM cleavage. The capsid (C) protein packages viral RNA and initiates particle formation. NS1 supports replication, immune evasion, and viral assembly. NS2A is involved in replication, polyprotein processing, and cytopathogenesis. NS2B, as a cofactor for NS3, forms a protease complex crucial for polyprotein processing and immune suppression. NS4A modulates host membranes and promotes viral protein oligomerization for replication. NS4B dimerizes, interacts with NS5, and helps form the replication complex (Nasar et al., 2020; Nath et al., 2024). NS3 and NS5 proteins have diverse enzymatic activities. NS5 contains a methyltransferase domain (N-terminal) for mRNA capping and an RNA-dependent RNA polymerase (RdRp) domain (C-terminal) for genome replication. NS5 is highly conserved among the four DENV serotypes, making it a promising target for anti-dengue drug development due to the absence of similar RdRp activity in human enzymes (Shimizu et al., 2019; Coulerie et al., 2013). The NS3 protease, consisting of NS2B and NS3, is crucial for DENV replication, functioning as a trypsin-like serine protease, with catalytic residues His51, Asp75, and Ser135. As a result, disrupting NS3 protein proves fatal to the virus, underscoring its potential as a key target for antiviral drug development (Tomlinson et al., 2009).

Several experimental studies have targeted NS3 and NS5 proteins to combat DENV infection. For example, - Abdullah et al. used computational methods to identify Zileuton, trimethadione, and linalool as novel NS3 inhibitors, with IC_50_ values of 3.3 mM, 25.97 mM, and 1.12 mM. They further proposed Ziltri and zilool based on docking results (Abdullah et al., 2023). Likewise, Balasubramanian et al. identified curcumin as a DENV2 NS2B/NS3 protease inhibitor, synthesizing analogs (CC1-CC5) with IC_50_ values between 36.23 and 66.01 μM, EC_50_ values between 8.07 and 29.25 μM, and CC50 values between 25.50 and 87.40 μM (Balasubramanian et al., 2019). Salleh et al. investigated 21 Malaysian medicinal plants and found that *Dryobalanops aromatica* methanol extract showed 99.7% inhibition at 200 μg/mL with an IC50 of 0.30 μg/mL targeting NS2B-NS3 protease (Salleh et al., 2019). Shimizu et al. identified RK-0404678 as a potent DENV2 NS5 RNA polymerase inhibitor with an EC_50_ of 6.0 μM, screened from 16,240 compounds (Shimizu et al., 2019). Jarerattanachat et al. identified Isoquercitrin as a dual-binding NS5 Methyltransferase inhibitor, effectively suppressing DENV with minimal toxicity (CC50 > 20 μM) (Jarerattanachat et al., 2023). However, only a few of these antiviral candidates have advanced to clinical trials, highlighting the need for novel DENV-targeted treatments.

In this concern, combining computational approaches with experimental studies offers a more effective strategy for developing antivirals against viral structural and non-structural proteins, expediting drug discovery. For example, - Indu et al. screened 7,000 phytocompounds against DENV proteins, identifying astragaloside II, III, and IV as potential inhibitors based on strong binding energies, which were further tested in Vero cell line (Indu et al., 2021). Similarly, Khan et al. assessed diterpenoids against dengue viral proteins (Envelope, NS1, NS3, NS5) using molecular docking, dynamics simulation, and network pharmacology (Khan et al., 2021). Cabarcas-Montalvo et al. docked 210,903 PubChem molecules against NS2B/NS3 and tested the top 5 candidates in antiviral assays (Cabarcas-Montalvo et al., 2016). In another study, Mirza et al. screened 18 million compounds from the ZINC database against NS3 protease using various *in silico* methods and tested 4 potent compounds through *in vitro* studies (Mirza et al., 2018). Furthermore, machine learning techniques (MLTs) have been widely applied in drug development. For example, - Gupta et al. developed DDPM, an early diagnostic model using MLTs to aid in dengue diagnosis and prognosis (Gupta et al., 2023). Similarly, Natali et al. used MLTs to identify rare antibody sequences capable of neutralizing pathogens (Natali et al., 2024). In this context, our group has developed several machine learning-based antiviral prediction tools utilizing quantitative structure–activity relationship (QSAR) information of molecules and peptides. Quantitative Structure–Activity Relationship (QSAR) is a computational modeling technique employed to establish relationships between the structural or physicochemical properties of chemical compounds and their biological activities. The fundamental assumption of QSAR is that variations in molecular structure lead to differences in biological behavior, including key pharmacokinetic parameters such as absorption, distribution, metabolism, excretion, and toxicity (ADMET) (Kwon et al., 2019). These include AVCpred for prediction and design of antiviral compounds (Qureshi et al., 2017), AVPpred for prediction of highly effective antiviral peptides (Thakur et al., 2012), AVP-IC50Pred for prediction of peptide antiviral activity in terms of half maximal inhibitory concentration (IC50) (Qureshi et al., 2015), and HIVprotI for prediction and design of HIV proteins inhibitors (Rajput and Kumar, 2022), among others. Furthermore, we have created MLT-based platforms such as anti-Flavi (Rajput and Kumar, 2018), anti-Nipah (Rajput et al., 2019), and anti-corona (Rajput et al., 2021a) to predict antiviral compounds against various pathogens. Additionally, we created a database of repurposed drugs effective against 23 epidemic and pandemic-associated viruses (Rajput et al., 2021b).

Recently, we developed the Anti-Dengue platform to predict repurposed drugs targeting DENV based on their IC_50_/pIC_50_ values (in μM) using various MLTs. However, this platform focuses on the entire virus without specifying the targeted site of the drug (Gautam et al., 2024). To address this gap, we introduce a new machine-learning-driven pipeline named “i-DENV”, which enables the identification of inhibitors targeting NS3 and NS5 proteins of DENV. The platform employs diverse MLTs (Support vector machine (SVM), Random Forest (RF), k-nearest neighbor (kNN), Artificial neural network (ANN), Extreme Gradient Boosting (XGBoost) and Deep Neural Network (DNN)). Using our top-performing models, we screened the DrugBank database to predict promising repurposed drug candidates for NS3 and NS5 proteins. Selected drug candidates underwent additional support through molecular docking, confirming strong binding affinities. Overall, this study contributes to the discovery of antiviral drugs specifically targeting NS3 and NS5 proteins, offering potential therapeutic benefits in combating DENV infection.

## Methodology

**FIGURE 1:**
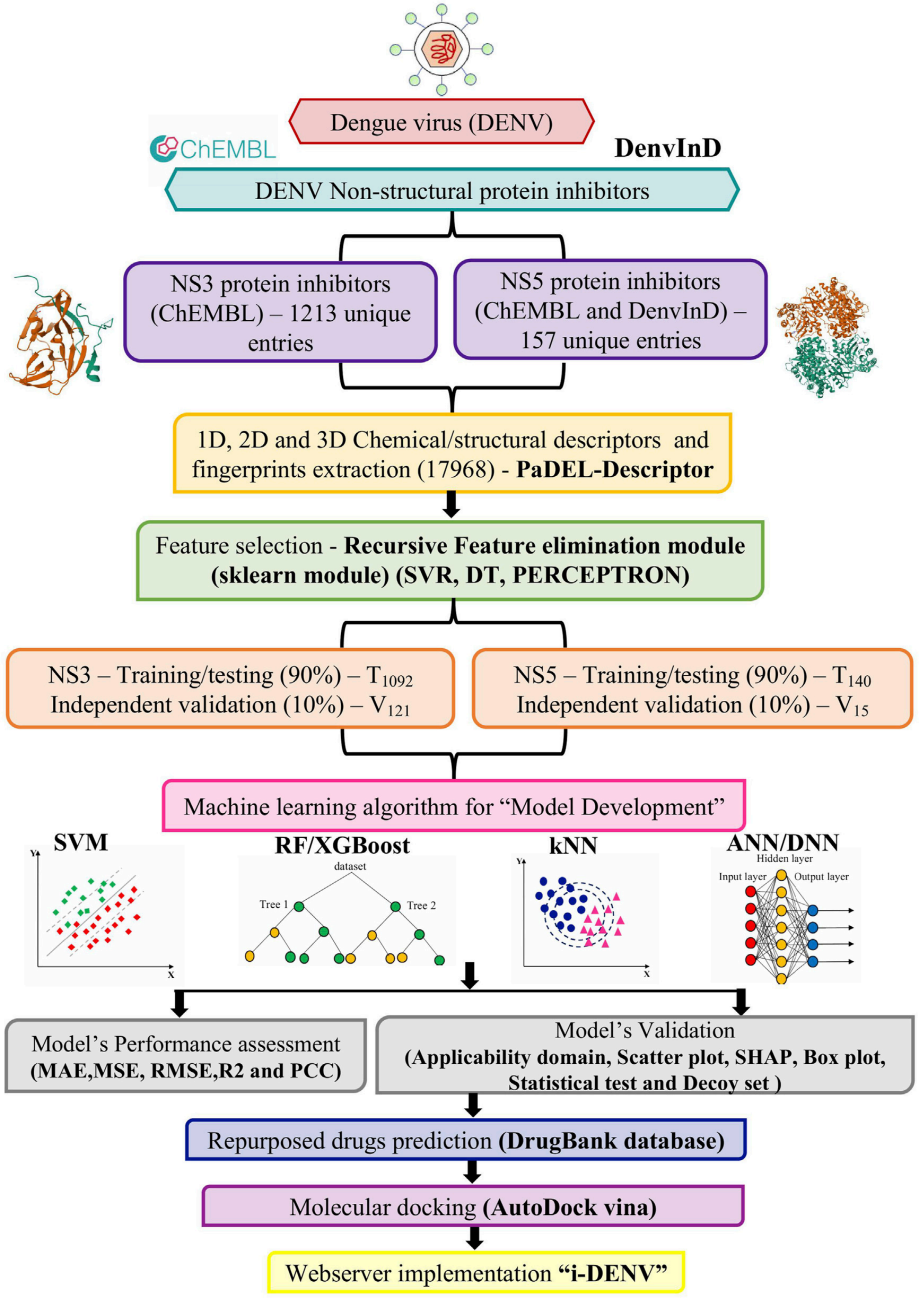
Curated data from ChEMBL and DenvInD, resulting in 1213 unique entries targeting the NS3 protein and 157 unique entries targeting the NS5 protein. Using PaDEL software, descriptors in one-dimensional, two-dimensional, and three-dimensional formats were computed. Recursive feature elimination method from the sklearn module was employed for feature selection. The data was then divided into training and testing datasets, and various MLTs were applied. Model performance was assessed using MAE, MSE, RMSE, R2, and PCC, and validated with applicability domain, scatter plot, chemical clustering, box plot, statistical test such as paired t-tests or Wilcoxon signed-rank tests and decoy set analysis. Potential repurposed drugs were identified through an analysis of the DrugBank database using the best-developed models for both NS3 and NS5 inhibitors. For further validation, the top predicted drugs were docked against NS3 and NS5 proteins using AutoDock Vina. The best-performing models were integrated into the web server “i-DENV”.

For developing the “i-DENV” predictive algorithm, the overall workflow is given in Figure 1.

## Data collection

For the predictor, experimentally validated compounds were taken from the CHEMBL and DenvInD databases. CHEMBL provides data on bioactive molecules with drug-like properties, integrating chemical, bioactivity, and genomic data to aid in developing new pharmaceutical treatments (Mendez et al., 2019; Davies et al., 2015). DenvInD is a comprehensive database encompassing known inhibitors targeting potential therapeutic targets of the DENV (Dwivedi et al., 2021).

## Steps for data retrieval

• ChEMBL provided information on 1,372 compounds for NS3 and 67 for NS5 protein, while DenvInD offered data on 95 compounds specifically targeting NS5 protein (Mendez et al., 2019) (Davies et al., 2015) (Dwivedi et al., 2021).
• The NS3 and NS5 dataset underwent a filtering process to extract inhibitors with EC_50_/IC_50_ values and corresponding SMILES representations. Subsequently, redundant entries were removed.
• Finally, we identified 1213 and 155 unique entries targeting NS3 and NS5 protein, respectively.
• The unique entries’ half-maximal inhibitory concentration (IC_50_), given in molar concentration, was transformed into pIC_50_ using the equation pIC_50_ = -log10(IC_50_). This transformation facilitates easier interpretation and comparison of drug potency by presenting the data on a consistent and more intuitive logarithmic scale.

Supplementary Table S1 provides the dataset of drugs and inhibitors used to develop the model for NS3 and NS5 protein.

## Format conversion

The compound structures from the NS3 and NS5 dataset were converted from SMILES to structure-data file (3D-SDF) format using Open Babel version 3.1.1 (O’Boyle et al., 2011). These converted files were subsequently employed as input to extract chemical descriptors and fingerprints.

## Computation of molecular descriptors and fingerprints

To develop QSAR based predictive models for the NS3 and NS5 proteins of DENV, we used PaDEL software to compute molecular descriptors and fingerprints (Yap, 2011). We computed 17,968 chemical descriptors for each molecule in the NS3 and NS5 protein dataset. Descriptors are numeric representations of molecular features, while fingerprints capture structural fragments, connectivity, bonds, and functional groups using binary sequences, where 1 denotes presence and 0 denotes absence (Grisoni et al., 2018). Descriptors and fingerprints are essential for analyzing drugs and chemicals, as they help assess their QSAR (Perkins et al., 2003).

## Feature selection

Feature selection is the process of selecting the most important features from a larger set to enhance model performance and interpretability (Taghizadeh et al., 2022). Feature selection used the Recursive Feature Elimination (RFE) module from scikit-learn with Perceptron, Support Vector Regression (SVR), and Decision Tree (DT) methods. The goal was to identify the top 50, 100, 150, and 200 features for each dataset. All selected features were then used as input for MLTs on the NS3 and NS5 datasets (Lin et al., 2012) (Gholami et al., 2012).

## Randomized dataset creation

Random selection processes were used to create a training/testing (TT) and independent validation (IV) dataset. To achieve this, 10% of the total data were randomly allocated as an IV dataset, with the remaining 90% utilized for the TT dataset. This process was repeated 5 times to get different 5 sets of TT and IV dataset. The NS3 dataset comprised 1213 molecules - T_1092_ and V_121_, while the NS5 dataset consisted of 155 molecules - T_140_ and V_15_.

## Ten-fold cross-validation

Ten-fold cross-validation is employed to assess the performance of a machine learning model. This method entails dividing the dataset randomly into ten equal subsets, or “folds”. The model undergoes ten rounds of training and evaluation, where each round uses a different fold as the validation set and the remaining nine folds as the training set. This systematic approach ensures that each data point is validated exactly once. Afterward, the performance metrics obtained from each round are averaged to provide a more accurate evaluation of the model’s performance.

## Model performance assessment

We evaluated the model’s performance using metrics such as Mean Absolute Error (MAE), Mean Squared Error (MSE), Root Mean Squared Error (RMSE), Coefficient of Determination (R2), and Pearson’s Correlation Coefficient (PCC or R). These metrics were calculated using the formulas provided below-

$$PCC=\frac{n\sum_{n=1}^{n}\;E_{i}^{act}E_{i}^{pred}-\sum_{n=1}^{n}\;E_{i}^{act}\sum_{n=1}^{n}\;E_{i}^{pred}}{\sqrt{n\sum_{n=1}^{n}\;{{\left(E_{i}^{act}\right)}^{2}-\left({\sum_{n=1}^{n}\;E}_{i}^{act}\right)}^{2}}-\sqrt{n\sum_{n=1}^{n}\;{{\left(E_{i}^{pred}\right)}^{2}-\left({\sum_{n=1}^{n}\;E}_{i}^{pred}\right)}^{2}}}$$

$$MAE=\frac{1}{n}\sum_{n=1}^{n}\;\left|E_{i}^{pred}-E_{i}^{act}\right|$$

$$RMSE=\sqrt{\frac{1}{n}\sum_{n=1}^{n}\;{\left(E_{i}^{pred}-E_{i}^{act}\right)}^{2}}$$

Where, ‘n' represents the size of the dataset, ‘Eact’ denotes the actual values, and ‘Epred’ corresponds to the predicted values.

## Robustness assessment and comparative analysis using statistical tests

To ensure the robustness of predictive models, 95% confidence intervals (CIs) were computed for key performance metrics (PCC, MAE, MSE, RMSE, and *R* ^2^) using 10-fold cross-validation across all machine learning techniques (MLTs). Statistical significance of performance differences among models was assessed through pairwise comparisons of these metrics. The Shapiro-Wilk test was used to evaluate normality, determining the choice of statistical tests: a paired t-test for normally distributed data or the Wilcoxon signed-rank test for non-normally distributed data. Additionally, box plots were generated using Seaborn and Matplotlib libraries to visualize the distribution and variability of performance metrics across different models (Hazra, 2017; Shapiro and Wilk, 1965; Rosner et al., 2006; Ross and Willson, 2017; Williamson et al., 1989).

## SHAP analysis

SHAP (SHapley Additive exPlanations) was employed to analyze feature importance in the best predictive Support Vector Machine (SVM) model for pIC_50_ prediction targeting the NS3 and NS5 protein. A Support Vector Regression (SVR) model with optimized hyperparameters was trained using 10-fold cross-validation. Features were standardized using StandardScaler, and SHAP values were computed using a KernelExplainer with a k-means-selected background dataset of 50 representative training samples. The SHAP values were aggregated across all folds to generate a comprehensive beeswarm and summary plot, highlighting the most influential features (Lundberg and Lee, 2017).

## Applicability domain analysis

In addition to model performance, accuracy for new predictions is crucial. Applicability domain (AD) analysis establishes the model’s boundaries to ensure reliable predictions. A query molecule’s chemical properties must fall within the AD of the trained model to ensure precise predictions (Kar et al., 2018).

To assess this, William’s plot was employed using a leverage approach for NS3 and NS5 proteins, illustrating the relationship between leverage and standardized residuals. The leverage threshold (h*) is:

$$\text{Leverage threshold }\left(h*\right)=3\left(p+1\right)n$$

where p - the number of descriptors used in developing the model. n is the number of compounds used in the training dataset.

The leverage threshold (h∗) is a key component of AD analysis, used to identify influential or outlier compounds in a QSAR model.

Leverage (h) quantifies a compound’s influence on the model. If h > h∗, the compound is flagged as an outlier or highly influential, indicating it may fall outside the AD and require further evaluation. Compounds within h∗ are considered reliably predicted by the model. The factor 3 in the formula is an empirically chosen value commonly used in QSAR modeling to set a practical threshold (Khosrokhavar et al., 2010).

To affirm the models’ effectiveness, scatter plots were created to compare predicted pIC_50_ values with actual pIC_50_ values for the top-performing models in both NS3 and NS5 datasets.

## Decoy sets analysis

Using the DecoyFinder 2.0 tool, Decoys were created for these drug candidates (Cereto-Massagué et al., 2012). We used a molecular weight-based method to create these decoys, utilizing the ZINC20 database, which includes 4.78 million drug-like molecules (Irwin et al., 2020).

Four distinct decoy datasets were created, containing 1213 decoys for the NS3 dataset and 155 for the NS5 dataset. These decoys, randomly generated to correspond to active drug candidates, were processed further through format conversion and molecular descriptor calculations to obtain their pIC_50_ values. A correlation analysis was then performed to measure the Pearson’s Correlation Coefficient (PCC), evaluating the relationship between the pIC_50_ values of the decoys and the actual pIC_50_ values of the corresponding active drug candidates.

## Chemical clustering analysis

We performed chemical clustering analysis using two methods: the ChemMine tool and t-distributed Stochastic Neighbor Embedding (t-SNE) visualization. In the ChemMine tool, molecular SMILES were used as input to assess drug heterogeneity, applying both multidimensional scaling (MDS) and binning clustering with a similarity threshold of 0.6 (Backman et al., 2011). For t-SNE, the dataset containing molecular descriptors and pIC_50_ values was loaded using Pandas and standardized with StandardScaler to ensure uniform feature scaling. t-SNE from sklearn. manifold was applied to reduce dimensionality to two components for visualization. A scatter plot was generated using matplotlib. pyplot, with pIC_50_ values as the color gradient to illustrate activity distribution (Van Der Maaten and Hinton, 2008).

## Drug repurposing

We used top-performing SVM and ANN models for both NS3 and NS5 proteins to identify potent repurposed drug candidates. We then screened 2,150 approved drugs from the Drugbank database to identify promising candidates (Knox et al., 2024). As a preliminary step, transformed the file format and calculated molecular descriptors of these drugs with PaDEL software. These descriptors were then used to identify potential repurposed drug candidates for DENV.

## Molecular docking

After identifying highly promising drug candidates targeting dengue NS proteins, the top 4 drugs in each category, based on 3D structure availability and predicted pIC_50_ values, were selected for molecular docking studies. For ligand preparation, the selected drugs 3D structures were acquired from PubChem in SDF format, converted to PDB format using PyMOL software, and then import the file in auto dock tools to convert it into PDBQT format (Eberhardt et al., 2021) (Trott and Olson, 2010).

To prepare the receptors, the 3D crystal structures of the DENV NS2B/NS3 protease (PDB ID: 2FOM) and NS5 polymerase (PDB ID: 4V0Q) were retrieved in PDB format from the RCSB Protein Data Bank. These structures were then loaded into Discovery Studio to remove the original ligands—glycerol (GOL) from the NS2B/NS3 protease and S-adenosyl-l-homocysteine (SAH) from the NS5 polymerase.

Prior to docking analyses, the 3D protein structures were refined by removing extraneous ions, ligands, and non-essential water molecules. Polar hydrogen atoms and Kollman charges were added to the receptor proteins. The refined structures were then saved in PDBQT format for the subsequent docking experiments.

To perform the docking simulations, AutoDock Vina (version 1.1.2) with default settings was used to create grid boxes for the dengue NS3 and NS5 proteins. Nine optimal docking conformations were then generated for both proteins and the inhibitor molecules (Eberhardt et al., 2021) (Trott and Olson, 2010).

The exhaustiveness parameter was set to 8 to compute the minimum binding affinity between proteins and ligands. Subsequently, the interactions were then analyzed and visualized using PyMOL and Discovery Studio Visualizer (DSV) (Rigsby and Parker, 2016) (Biovia et al., 2016).

## Web server development

The “i-DENV” web server, designed to predict a chemical’s pIC_50_ and IC_50_ (μM) for inhibiting DENV, integrates the most effective SVM models for NS3 and NS5 proteins. It is built using the LAMP software stack, which includes Linux (OS), Apache (web server), MySQL (database), and PHP (scripting language). The system runs on Ubuntu 20.04.6 LTS (Focal Fossa), a Debian-based Linux distribution, with Apache 2.4.41 as the web server and MySQL 8.0.40-0ubuntu0.20.04.1 on a 64-bit Linux system. For server-side scripting, it utilizes PHP 7.4.3-4ubuntu2.28 (CLI), incorporating Zend Engine v3.4.0 and Zend OPcache v7.4.3-4ubuntu2.28 for optimized performance.

The “i-DENV” web server’s user interface is built with HTML, CSS, and PHP, while the backend is powered by Python, Perl, and JavaScript to handle computations and data processing. To improve accessibility, it includes dedicated “Help” and “FAQs” pages, ensuring users have guidance and support while using the platform.

## Results

### Feature selection

For the NS3 dataset, top performing SVM and ANN models used 200 features selected by SVR, including KRFP1702, FPSA-1, and others. Supplementary Table S2 presents the top 200 features identified through SVR, DT, and Perceptron using the RFE module. For the NS5 dataset, the top SVM and ANN models used 100 features selected by SVR, including SubFP151, KRFPC4060, KRFPC1634, KRFPC1670, ExtFP453 and others. Supplementary Table S2 presents the top 100 features identified through SVR, DT, and Perceptron using the RFE module.

### Assessment of the effectiveness of MLTs-Based QSAR models

To identify potential inhibitors for DENV, we developed prediction models using six MLTs and datasets with 200 features for NS3 and 100 features for NS5 protein.

To assess the performance of the developed QSAR models, Diverse statistical metrics were employed like MAE, MSE, R2, RMSE, and PCC. PCC measures how strongly predicted pIC_50_ values align with actual pIC_50_ values, ranging from −1 (negative correlation) to +1 (positive correlation), with 0 indicating no correlation. *R* ^2^ values measure how well data aligns with a statistical model. A value of 1 indicates a perfect fit, while 0 indicates no fit at all. MAE shows how close predicted values are to actual values, while RMSE measures the average magnitude of errors. Lower values of both MAE and RMSE indicate better model performance and *vice versa*.

For the DENV NS3 dataset, the best SVM and ANN models achieved PCC values of 0.857 and 0.862 on the TT dataset and 0.870 and 0.894 on the IV dataset. Similarly, for the DENV NS5 dataset, the top SVM and ANN models demonstrated PCC values of 0.982 and 0.964 on the TT dataset and 0.970 and 0.977 on the IV dataset, respectively. The performance metrics of best performing models developed for NS3 and NS5 Protein using various MLTs and Feature Selection methods on TT and IV Datasets are given in Table 1. The detailed information about best predictive developed models are mentioned in Supplementary Table S3.

**TABLE 1:** Performance Metrics of best performing models developed for NS3 and NS5 Protein using various MLTs and Feature Selection method on TT and IV Datasets.

Algorithm	Feature selection	Model parameters	Dataset	MAE	MSE	RMSE	*R* ^2^	PCC
NS3								
SVM	SVR	gamma:0.0001 C:100	**T**_**1092**_	**0.191**	**0.065**	**0.255**	**0.733**	**0.857**
			**V**_**121**_	**0.195**	**0.074**	**0.272**	**0.756**	**0.870**
	Perceptron	gamma:0.001 C:10	T_1092_	0.239	0.099	0.315	0.593	0.771
			V_121_	0.212	0.087	0.295	0.713	0.844
	DT	gamma:0.005 C:1	T_1092_	0.255	0.118	0.343	0.517	0.719
			V_121_	0.215	0.097	0.311	0.680	0.833
RF	SVR	n:400	T_1092_	0.264	0.124	0.352	0.492	0.703
			V_121_	0.213	0.093	0.305	0.693	0.851
	Perceptron	n:500	T_1092_	0.275	0.130	0.361	0.467	0.684
			V_121_	0.225	0.102	0.319	0.663	0.832
	DT	n:400	T_1092_	0.266	0.124	0.350	0.475	0.705
			V_121_	0.218	0.103	0.321	0.66	0.825
kNN	SVR	k:3	T_1092_	0.251	0.119	0.346	0.511	0.725
			V_121_	0.250	0.131	0.363	0.565	0.756
	Perceptron	k:7	T_1092_	0.267	0.127	0.356	0.480	0.695
			V_121_	0.227	0.105	0.325	0.651	0.811
	DT	k:5	T_1092_	0.270	0.131	0.362	0.464	0.690
			V_121_	0.233	0.109	0.330	0.639	0.799
ANN	SVR	solver:lbfgs activation: identity	**T**_**1092**_	**0.192**	**0.064**	**0.253**	**0.738**	**0.862**
			**V**_**121**_	**0.190**	**0.070**	**0.265**	**0.781**	**0.894**
	Perceptron	activation:logistic	T_1092_	0.249	0.108	0.329	0.556	0.751
			V_121_	0.216	0.086	0.293	0.716	0.844
	DT	activation: logistic	T_1092_	0.275	0.135	0.365	0.426	0.682
			V_121_	0.241	0.111	0.332	0.634	0.798
XGBoost	SVR	n_estimators = 300, max_depth = 3, learning_rate = 0.141	T_1092_	0.249	0.111	0.334	0.544	0.738
			V_121_	0.222	0.087	0.296	0.710	0.849
	Perceptron	n_estimators = 300, max_depth = 7, learning_rate = 0.058	T_1092_	0.272	0.132	0.362	0.448	0.678
			V_121_	0.212	0.089	0.298	0.705	0.842
	DT	n_estimators = 200, max_depth = 3, learning_rate = 0.104	T_1092_	0.259	0.119	0.344	0.514	0.718
			V_121_	0.225	0.096	0.31	0.681	0.830
NS5								
SVM	SVR	gamma:0.0001 C:400	**T**_**140**_	**0.135**	**0.049**	**0.197**	**0.954**	**0.982**
			**V**_**15**_	**0.138**	**0.044**	**0.210**	**0.94**	**0.970**
	Perceptron	gamma:0.0005 C:400	T_140_	0.222	0.105	0.310	0.884	0.953
			V_15_	0.24	0.137	0.370	0.814	0.904
	DT	gamma:0.005 C:10	T_140_	0.429	0.399	0.591	0.632	0.802
			V_15_	0.420	0.446	0.668	0.395	0.713
RF	SVR	n:400 depth: 12	T_140_	0.399	0.324	0.544	0.659	0.840
			V_15_	0.340	0.211	0.46	0.713	0.863
	Perceptron	n:300	T_140_	0.360	0.294	0.513	0.680	0.852
			V_15_	0.288	0.180	0.425	0.755	0.873
	DT	n:200 depth: None leaf:1	T_140_	0.424	0.388	0.601	0.560	0.799
			V_15_	0.367	0.308	0.555	0.582	0.771
kNN	SVR	k:3	T_140_	0.343	0.235	0.468	0.727	0.889
			V_15_	0.292	0.144	0.380	0.804	0.901
	Perceptron	k:3	T_140_	0.335	0.235	0.468	0.753	0.895
			V_15_	0.360	0.232	0.481	0.686	0.833
	DT	k:3	T_140_	0.508	0.499	0.687	0.446	0.739
			V_15_	0.563	0.609	0.781	0.173	0.663
ANN	SVR	solver: lbfgs activation: identity learning: invscaling	**T**_**140**_	**0.159**	**0.073**	**0.271**	**0.928**	**0.964**
			**V**_**15**_	**0.160**	**0.048**	**0.219**	**0.935**	**0.977**
	Perceptron	solver: lbfgs activation: logistic learning: adaptive	T_140_	0.255	0.119	0.345	0.884	0.942
			V_15_	0.337	0.238	0.488	0.710	0.854
	DT	solver: lbfgs activation: tanh learning: adaptive	T_140_	0.532	0.505	0.711	0.508	0.762
			V_15_	0.507	0.592	0.769	0.197	0.708
XGBoost	SVR	n_estimators = 300, max_depth = 3, learning_rate = 0.078	T_140_	0.334	0.22	0.444	0.766	0.889
			V_15_	0.388	0.274	0.523	0.628	0.818
	Perceptron	n_estimators = 300, max_depth = 3, learning_rate = 0.143	T_140_	0.335	0.243	0.458	0.678	0.846
			V_15_	0.313	0.200	0.447	0.729	0.870
	DT	n_estimators = 150, max_depth = 5, learning_rate = 0.038	T_140_	0.406	0.326	0.548	0.609	0.812
			V_15_	0.358	0.207	0.455	0.719	0.854

SVR, Support vector regression; DT, Decision tree; TT, Training or testing dataset; IV, Independent validation dataset; PCC, Pearson’s correlation coefficient, *R* ^2^ - coefficient of determination, MAE, mean absolute error; MSE, mean squared error; RMSE, root mean squared error. Bold values indicate the best-performing model(s) based on Pearson correlation coefficient (PCC) and coefficient of determination (R^2^) for each dataset.

The performance metrics of the DNN are provided in Supplementary Table S4.

### Robustness assessment and comparative analysis using statistical tests

The predictive performance of the machine learning models was assessed using 10-fold cross-validation, reporting key metrics (MAE, MSE, RMSE, *R* ^2^, and PCC) with 95% confidence intervals. SVM-SVR (gamma: 0.0001, C: 100) achieved the lowest error and highest predictive accuracy for NS3, with an MAE of 0.182–0.200, RMSE of 0.236–0.272, *R* ^2^ of 0.678–0.764, and PCC of 0.831–0.877. For NS5, SVM-SVR (gamma: 0.0001, C: 400) exhibited superior performance (MAE: 0.091–0.178, RMSE: 0.122–0.273, *R* ^2^: 0.928–0.979, PCC: 0.972–0.991), indicating highly accurate predictions.

Statistical analysis (Shapiro-Wilk test for normality, paired t-test/Wilcoxon signed-rank test) confirmed SVM as the best-performing model for both NS3 and NS5, significantly outperforming RF, kNN, and XGBoost across all metrics. ANN showed competitive performance, with no significant difference from SVM, while RF and ANN outperformed kNN and XGBoost in several comparisons.

**FIGURE 2:**
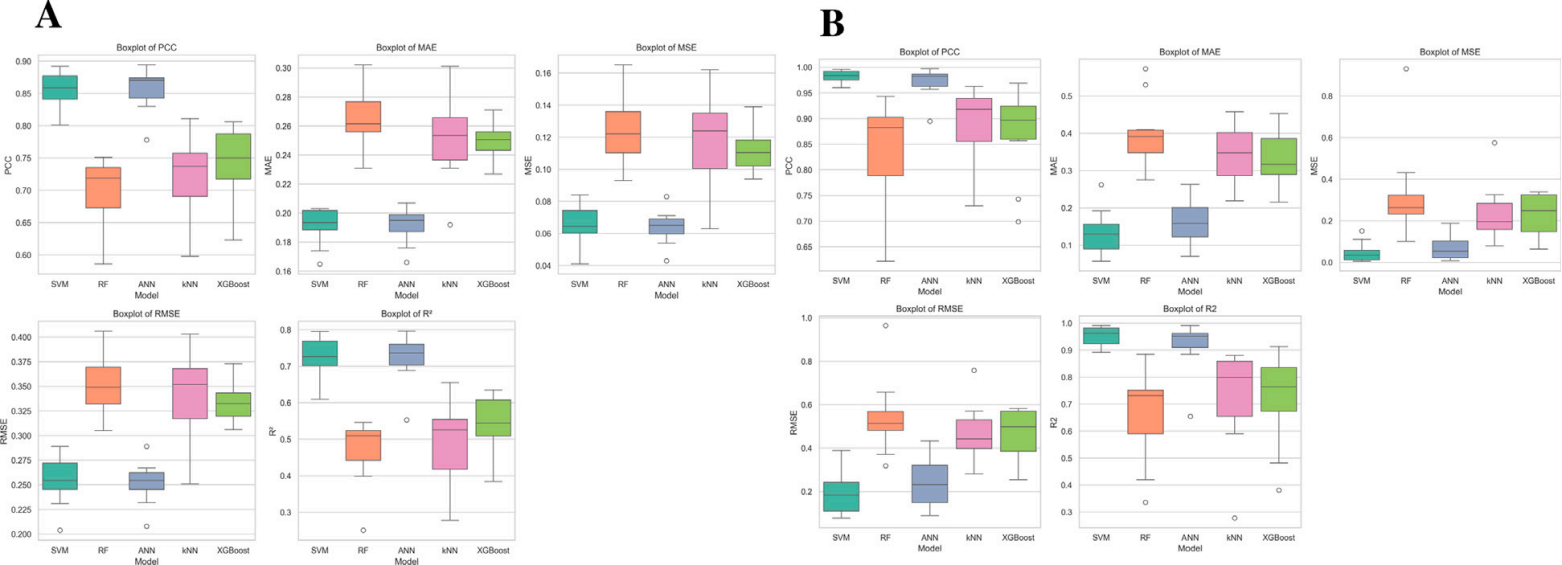
Performance comparison of five **(A)** NS3 and **(B)** NS5 machine learning models (SVM, RF, ANN, kNN, and XGBoost) using five evaluation metrics: PCC, MAE, MSE, RMSE, and *R* ^2^. Each boxplot illustrates the distribution of metric values across multiple runs, with the central line denoting the median, the box representing the interquartile range (IQR), and whiskers extending to 1.5 times the IQR. Outliers are depicted as individual points beyond this range.

Box plots as depicted in Figure 2 illustrated model performance variability, with SVM and ANN consistently achieving the best results (highest PCC and *R* ^2^, lowest errors), while RF showed high variability. kNN and XGBoost exhibited weaker predictive ability, with higher errors and lower correlation. These findings establish SVM as the most reliable model, followed by ANN, with kNN and XGBoost as the least effective.

The 95% confidence intervals and statistical analysis results (Shapiro-Wilk test for normality, paired t-test/Wilcoxon signed-rank test) are provided in the Supplementary Table S5.

### SHAP analysis

**FIGURE 3:**
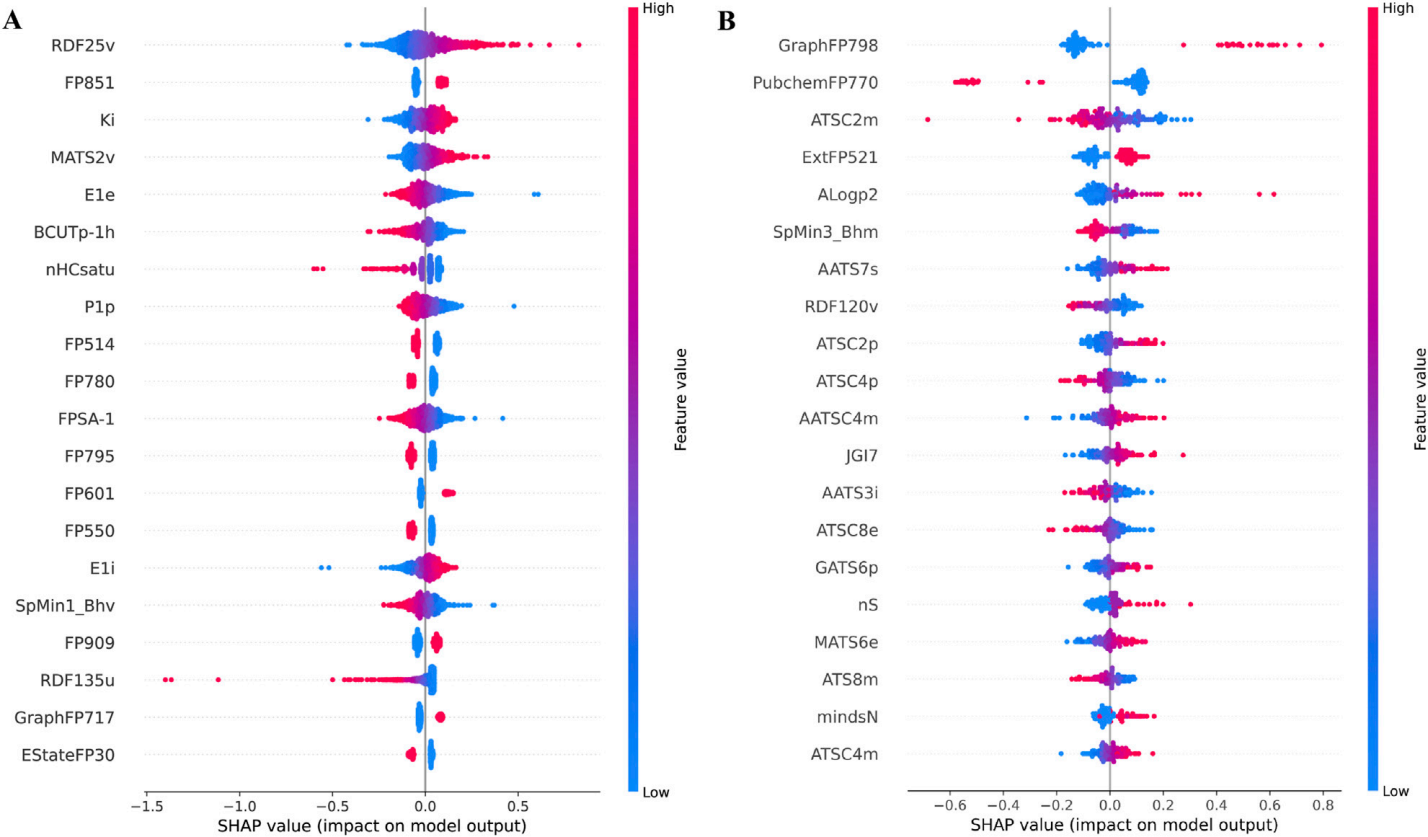
SHAP-Based Analysis of Key Features in the Best Predictive SVM Model for pIC50 Prediction: **(A)** Beeswarm Plot for NS3 Protein, **(B)** Beeswarm Plot for NS5 Protein. Each point represents a single sample (compound) and illustrates the impact of a feature on the model’s output. The x-axis indicates the SHAP value, representing the magnitude and direction of a feature’s contribution to the prediction. Features are ranked vertically by their overall importance, measured by the mean absolute SHAP value. The color gradient (blue to red) denotes the feature value for each sample, with blue indicating low values and red indicating high values. Points spread further from zero along the x-axis indicate greater influence on the prediction. These plots help visualize both the importance of each feature and the direction of its effect across the dataset.

SHAP is a theoretical approach that quantifies feature importance by assessing the impact of each feature on the model’s output. Figures 3A,B present Beeswarm plots, where each dot represents a SHAP value for a specific feature in an individual molecule. The x-axis denotes SHAP values, indicating both the direction and magnitude of each feature’s influence on model predictions. Positive SHAP values suggest that a feature enhances predicted activity, while negative values indicate a suppressive effect. The color gradient represents feature values, with red signifying higher values and blue representing lower values. Features that spread further along the x-axis exert greater influence on predictions. Supplementary Figures S1A and S1B provide mean absolute SHAP value plots, ranking features based on their overall contribution to model predictions. Figure 3A highlights RDF25v (Radial Distribution Function - 0.25, van der Waals volume-weighted) as the most influential descriptor, followed by FP851 (Fingerprint), Ki (K Global Shape Index, Ionization Potential-Weighted), and MATS2v (Moran Autocorrelation, Lag 2, van der Waals Weighted). Figure 3B highlights GraphFP798 (Graph-based Molecular Fingerprint), PubchemFP770 (Binary Molecular Fingerprint), and ATSC2m (Centered Broto-Moreau Autocorrelation, Lag 2, Mass-Weighted) as key predictive features. These findings underscore the significance of structural, electronic, and steric properties in driving model predictions.

### Applicability domain analysis

**FIGURE 4:**
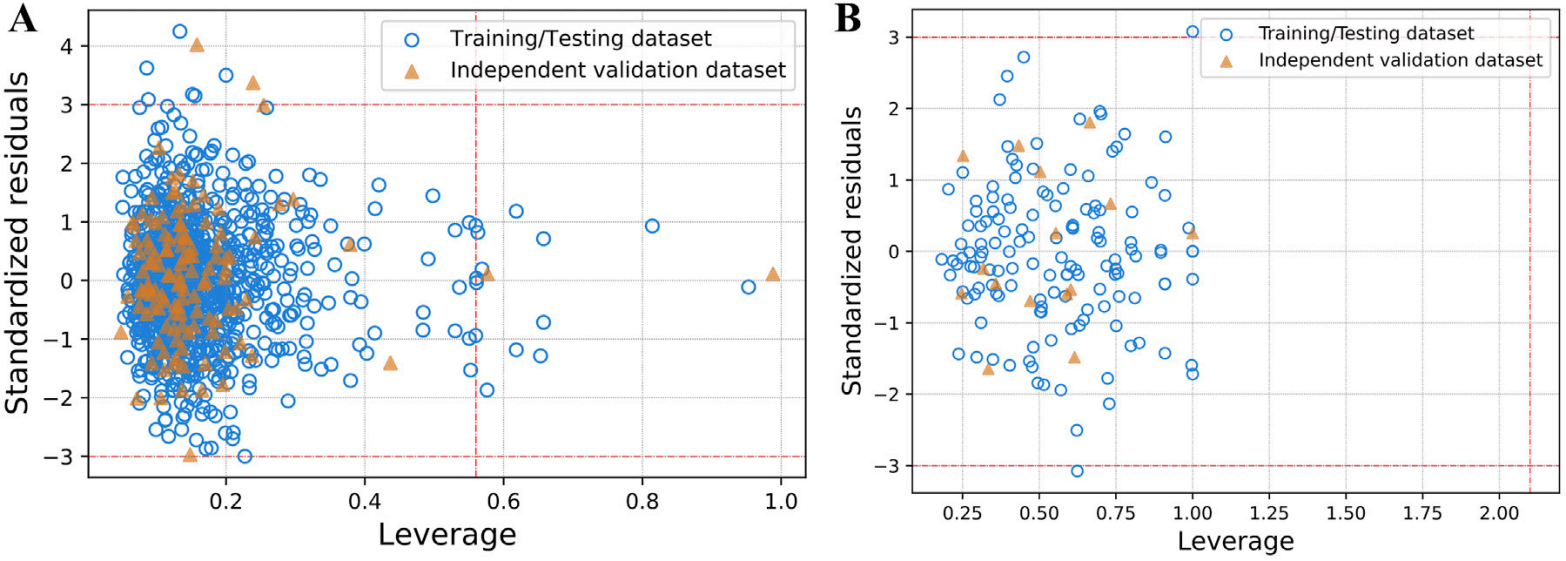
The applicability domain analysis using william plot for both **(A)** NS3 and **(B)** NS5 protein between the leverage and standardized residuals of the molecules. The x-axis represents the leverage values, indicating the influence of each compound on the model, while the y-axis shows the standardized residuals, reflecting the prediction error for each compound. Data points are color-coded: blue circles represent compounds from the training/testing dataset, and orange triangles represent those from the independent validation dataset. The horizontal dashed red lines at ±3 delineate the threshold for standardized residuals; points outside this range are considered outliers. The vertical dashed red line represents the critical leverage value (h*), beyond which compounds may be deemed influential and outside the model’s applicability domain. These plots help identify both outliers and structurally influential compounds, supporting the reliability and robustness of the developed models.

**FIGURE 5:**
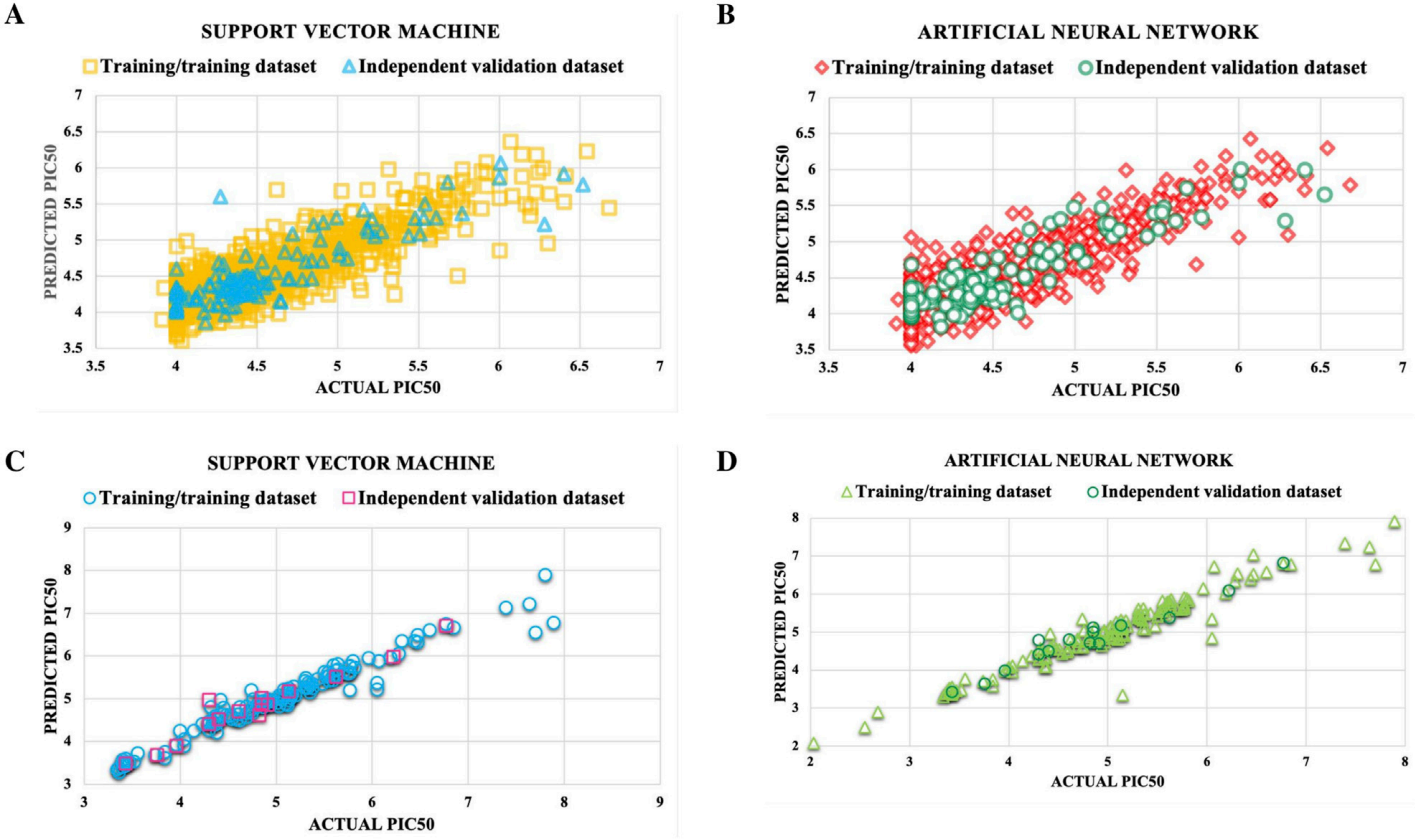
Reliability of the best predictive models for NS3 and NS5 was evaluated by generating scatter plots comparing the actual pIC50 values of molecules with their predicted values. The models assessed include: **(A)** SVM for NS3, **(B)** ANN for NS3, **(C)** SVM for NS5, and **(D)** ANN for NS5.

SVM model’s reliability for NS3 and NS5 proteins was validated via applicability domain analysis using William’s plot. Most data points fell within the h* values of 0.56 for NS3 and 2.1 for NS5, as depicted in Figure 4. This suggests that the SVM models for NS3 and NS5 proteins exhibit good reliability and generalization capacity for the given dataset. The presence of only a few points outside the critical h* threshold indicates minimal extrapolation, reinforcing the model’s robustness and predictive accuracy. These results validate the applicability of the SVM model for predicting biological activity related to NS3 and NS5 proteins. Furthermore, Figure 5 presents scatter plots of the best predictive models for NS3 and NS5 proteins, comparing the actual and predicted pIC50 values for both the TT and IV datasets. Scatter plots for the remaining models are provided in Supplementary Figures S2 and S3. The close clustering of data points inside the boundary line suggests developed models are highly accurate. Supplementary Table S6 contains the data utilized for the plotting William’s plot for NS3 and NS5 protein, while Supplementary Table S7 includes the actual and predicted pIC_50_ values for the scatter plots of NS3 and NS5 proteins.

### Verification with decoys

**FIGURE 6:**
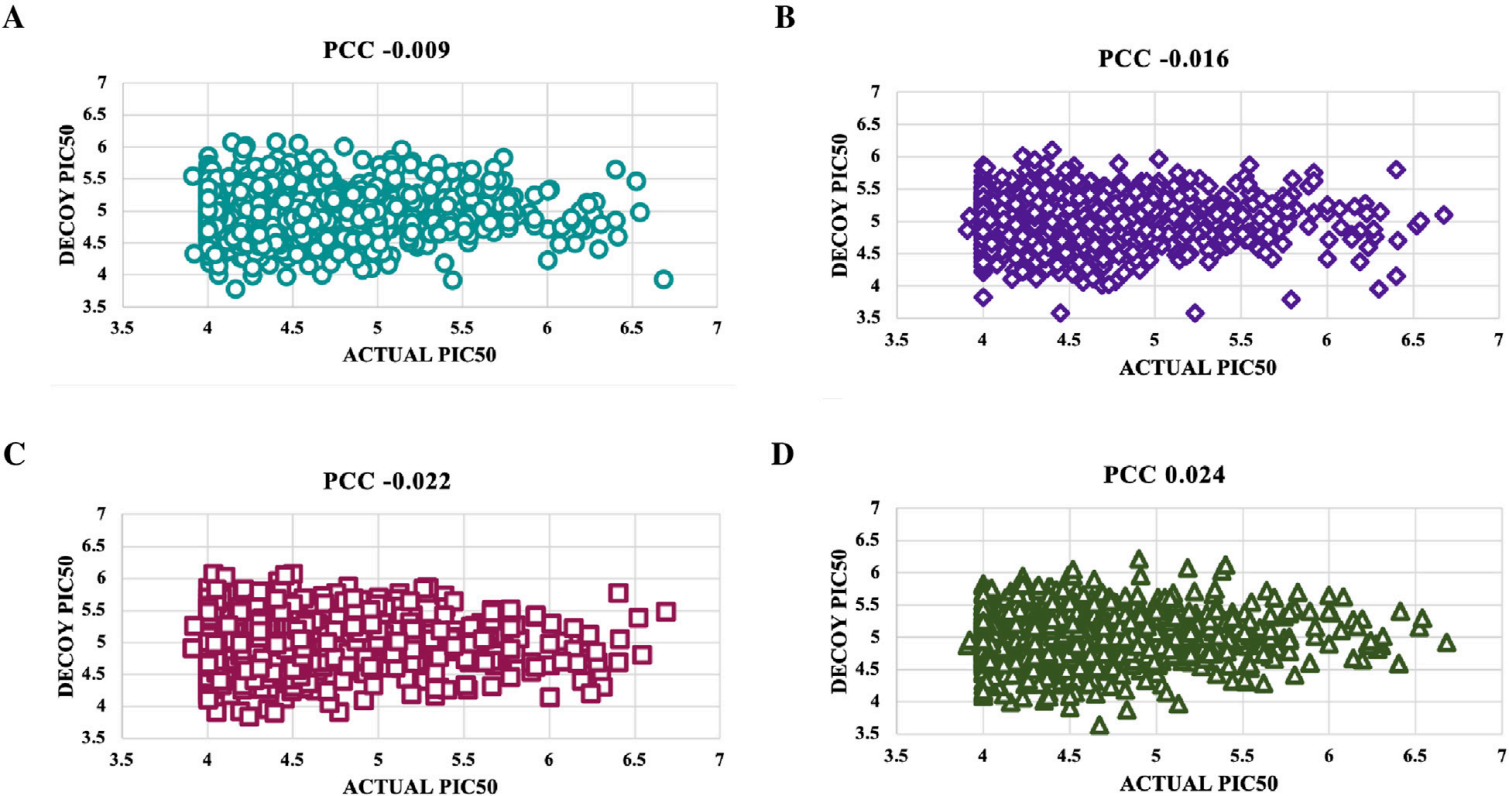
To validate the accuracy of the predicted models using the top-performing SVM model for NS3 protein, we created scatter plots. These plots were used to compare the actual pIC_50_ values with the decoy values from four distinct decoy sets: **(A)** Set 1, **(B)** Set 2, **(C)** Set 3, and **(D)** Set 4.

**FIGURE 7:**
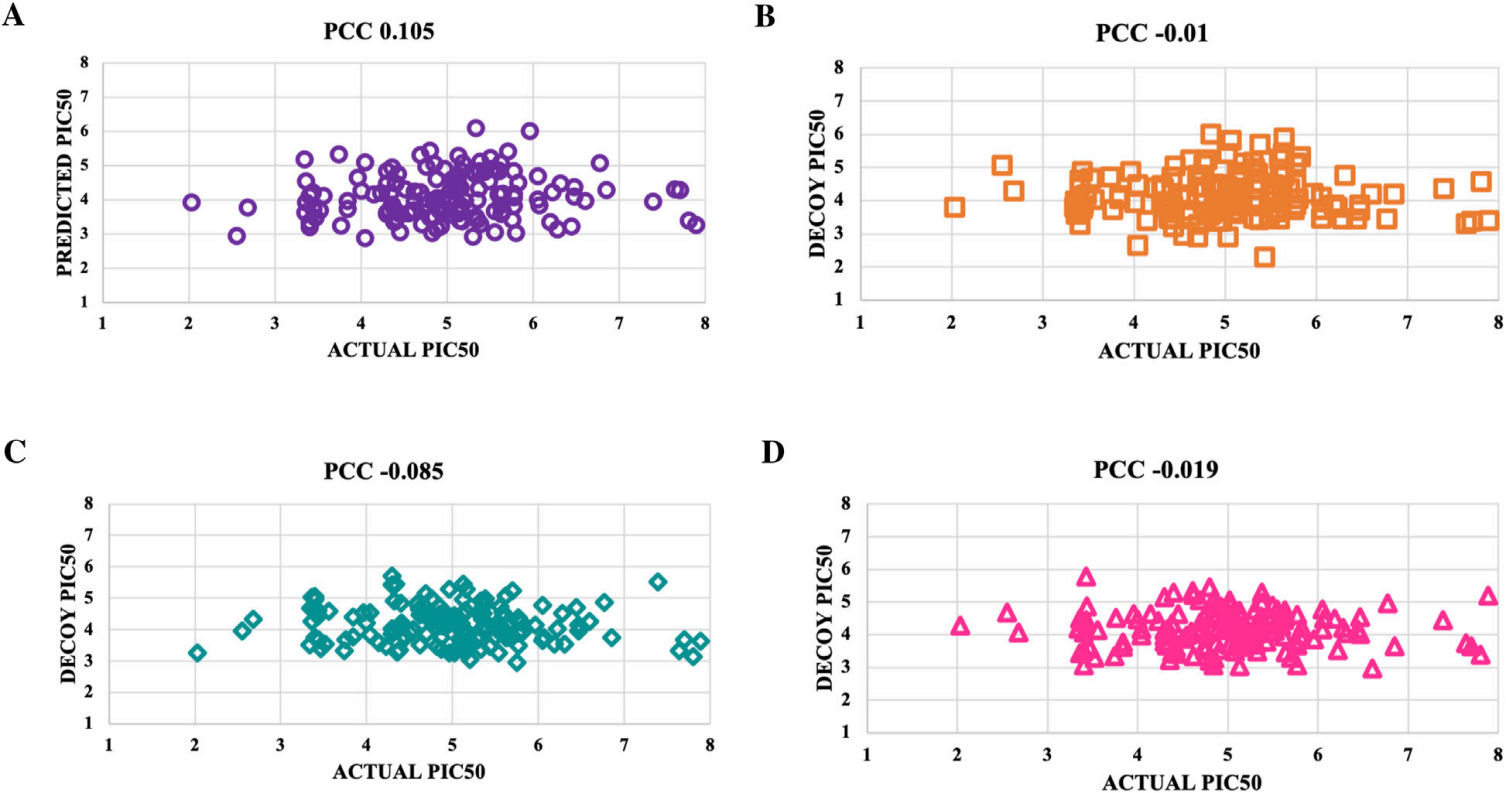
To validate the accuracy of the predicted models using the top-performing SVM model for NS5 protein, we created scatter plots. These plots were used to compare the actual pIC_50_ values with the decoy values from four distinct decoy sets: **(A)** Set 1, **(B)** Set 2, **(C)** Set 3, and **(D)** Set 4.

Decoys, which do not bind to targets, were used to validate the predictive model’s accuracy by comparing pIC_50_ values of four decoy sets with those of active molecules for NS3 and NS5 proteins (Supplementary Table S8). For the NS3 protein, Decoy sets 1 - 4 had PCC values of −0.009, −0.016, −0.022, and 0.024, respectively, shown in Figure 6. For the NS5 protein, Decoy sets 1 - 4 had PCC values of 0.105, −0.01, −0.085, and −0.019, respectively, shown in Figure 7.

### Analysis of chemical heterogeneity

**FIGURE 8:**
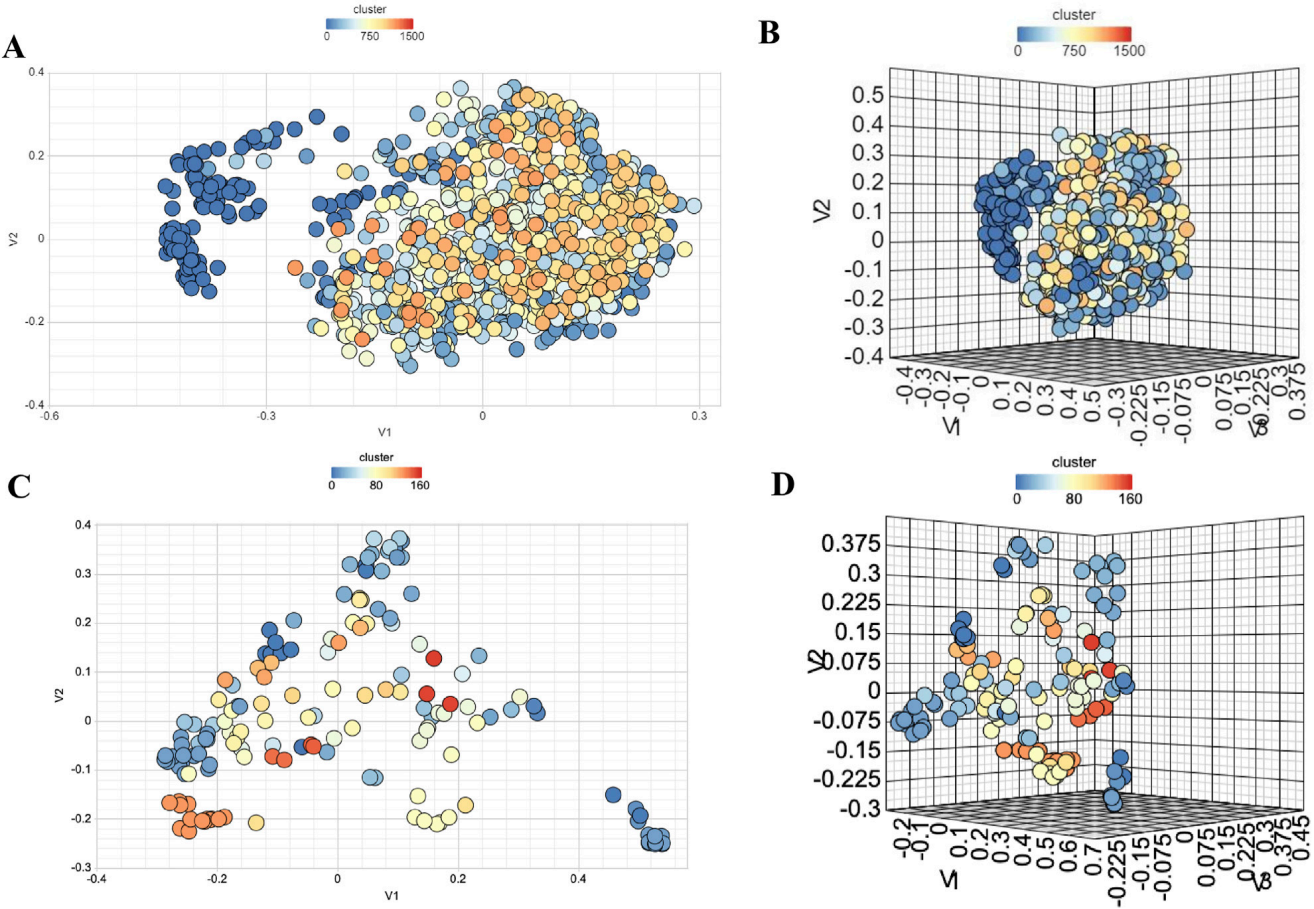
Chemical Diversity Analysis using **(A)** 2D MDS Plot for NS3, **(B)** 3D MDS Plot for NS3-Targeting Compounds, **(C)** 2D MDS Plot for NS5, and **(D)** 3D MDS Plot for NS5-Targeting Compounds.

Structural variability in NS3 and NS5 compounds was assessed through binning clustering analysis, which grouped NS3 compounds into 685 bins and NS5 compounds into 71 bins, using a similarity threshold of 0.6 (Supplementary Table S9). Additionally, 2D and 3D multidimensional scaling plots visualized dissimilarities among NS3 and NS5 compounds in chemical space, using a similarity threshold of 0.6, shown in Figure 8. t-Distributed Stochastic Neighbor Embedding (t-SNE) was applied to the standardized descriptor space to reduce dimensionality and visualize potential structure-activity relationships (SAR). The resulting distribution revealed distinct clusters of high bioactivity (yellow-green regions), suggesting potent molecular scaffolds, while lower-activity compounds (purple regions) were more dispersed, reflecting greater structural diversity as depicted in the Supplementary Figure S4. The gradual color transitions indicate that bioactivity is influenced by subtle variations in molecular descriptors rather than discrete clustering. Both these methods showed that compounds in both datasets exhibit diverse nature of chemicals.

### Prediction of promising repurposed drug candidates against NS3 and NS5 protein

The top SVM and ANN models for both NS3 and NS5 were used to predict repurposed drugs from the approved drugs category of the DrugBank database. The top candidates reported in the literature for both proteins are presented in Tables 2, while the remaining top candidates are provided in Supplementary Table S10.

**TABLE 2:** The top leading repurposed drug candidates predicted to be effective against NS3 and NS5 Protein of DENV with information such as drugbank ID, drug name, primary indication, predicted pIC_50_, and their current testing status.

DrugBank_ID	Target_Protein	Drug name	Primary indication	Predicted_pIC_50_ (ANN)	Predicted_pIC_50_ (SVM)	Status
DB01141	NS3	Micafungin	Treating candidemia, acute disseminated candidiasis, and other invasive *Candida* infections	7.818	6.743	Experimental
DB00035	NS3	Desmopressin	Synthetic vasopressin analog reduces water excretion in diabetes insipidus and nocturia	6.139	5.978	Computational
DB00362	NS3	Anidulafungin	Treatment of several types of *candida* infections	5.635	5.6	Experimental
DB00482	NS3	Celecoxib	NSAID for arthritis, pain, menstrual symptoms, and polyp reduction	5.476	5.263	Experimental and computational
DB08911	NS3	Trametinib	Kinase inhibitor for BRAF-mutated cancers like melanoma and non-small cell lung cancer, used alone or with dabrafenib	5.425	5.177	Experimental
DB00696	NS3	Ergotamine	Alpha-1 adrenergic agonist for treating migraines with or without aura and cluster headaches	5.375	5.105	Computational
DB00520	NS3	Caspofungin	Echinocandin treats various fungal infections	5.261	5.505	Experimental
DB00796	NS3	Candesartan cilexetil	For hypertension, left ventricular hypertrophy, and delaying diabetic nephropathy	5.15	5.102	Experimental
DB00091	NS3	Cyclosporine	Used in transplants and inflammatory conditions including ulcerative colitis, rheumatoid arthritis, and atopic dermatitis	5.144	5.452	Experimental
DB00471	NS3	Montelukast	Treating asthma, preventing exercise-induced bronchoconstriction, and managing seasonal allergic rhinitis	5.12	5.141	Experimental and computational
DB01017	NS5	Minocycline	Wide variety of infections	5.711	5.732	Experimental
DB00254	NS5	Doxycycline	Wide variety of infections	5.643	5.68	Experimental
DB01263	NS5	Posaconazole	Invasive infections by *Candida* species and Aspergillus species	5.529	5.506	Experimental
DB00997	NS5	Doxorubicin	Treating various cancers like AIDS-associated Kaposi’s Sarcoma and metastatic cancers	4.997	5.027	Experimental and computational
DB00686	NS5	Pentosan polysulfate	For relieving bladder pain or discomfort from interstitial cystitis	4.478	4.853	Experimental
DB00811	NS5	Ribavirin	For managing chronic HCV infection	3.526	3.737	Experimental

### i-DENV web server

To predict a query molecule’s inhibition efficiency, users upload or paste the SDF format of the query molecule. Results, including SMILES, pIC_50_, and IC_50_ (μM), are displayed in a table. Predictions may take a few minutes, and users can save or copy the job ID to check status later. The “i-DENV” web server is freely accessible at http://bioinfo.imtech.res.in/manojk/idenv/.

### Molecular docking of predicted promising inhibitors with NS3 and NS5 protein

Molecular docking finds the optimal conformation between a drug (ligand) and a protein (receptor) to achieve the minimum binding energy. In the NS3 and NS5 datasets, Delavirdine (DB00705) had the lowest binding energy of −8.3 kcal/mol against DENV NS2B/NS3 protease, as shown in Table 3, compared to Raltegravir (DB06817) at −8.1 kcal/mol, Rimexolone (DB00896) at −8.1 kcal/mol, and Fluoxymesterone (DB01185) at −7.9 kcal/mol. For DENV NS5 polymerase, Baloxavir marboxil (DB13997) demonstrated the lowest binding energy of −8.4 kcal/mol, detailed in Table 3. Conversely, Diphemanil (DB13720), Latamoxef (DB04570), and Cyclothiazide (DB00606), exhibited binding energies of −8.3, −7.4, and −7.3 kcal/mol, respectively.

**TABLE 3:** Docking interactions of selected drugs (inhibitors) with DENV NS2B/NS3 protease and DENV NS5 polymerase.

Ligand (Drugbank_ID)	Protein/Receptor (PDB_id)	Binding energies (kcal mol−1)	Interacting amino acid residues	Distance (Å)	Molecular interactions
Delavirdine (DB00705)	NS2B/NS3 (2FOM)	−8.3	Gly B:153 His B:51 Ser B:135 Leu B:128 Pro B:132	2.99 4.47 5.19 4.95 4.59 4.20 3.76 4.91 4.87 3.77	Van der waals Conventional Hydrogen bond Carbon hydrogen bond Pi-Sigma Pi-alkyl
Raltegravir (DB06817)		−8.1	Val B:52 Ile B:36 His B:51 Pro B:132 Arg B:54 Thr B:53 Ala B:56	4.19 3.39 5.78 5.84 5.40 5.43 5.88 4.76 4.83 4.75 5.98	Van der waals Conventional Hydrogen bond Carbon Hydrogen Bond Halogen (Fluorine) Pi- Cation Pi-Pi T- shaped Pi-alkyl
Rimexolone (DB00896)		−8.1	Ala B:56 Thr B:53 Val B:52 Ile B:36 Arg B:54 His B:51 Glu A:48	4.37 5.78	Van der waals Conventional Hydrogen bond alkyl
Fluoxymesterone (DB01185)		−7.9	Asn-B:152 Gly-B:153 Gly-B:151 Pro-B:132 Leu-B:128 Tyr-B:161	NA	Van der waals
Baloxavir_marboxil (DB13997)	NS5 (4V0Q)	−8.4	Phe-A:398 Ala-A:421 Val-A:402 Trp-A:418 Gln-A:602 Gly-A:604 Asn-A:492 Tyr-A:606 Val-A:603 Asn-A:405 Lys-A:401	6.42 7.57 5.28 4.05 7.37 3.27 5.12 5.14 3.54 6.68 5.85 6.67	Van der waals Conventional Hydrogen bond Carbon hydrogen bond Halogen (Fluorine) Sulfur-X Pi-Sulfur Pi-Alkyl
Diphemanil (DB13720)		−8.3	Lys-A:355 Arg-A:581 Pro-A:298	6.07 6.03 4.47	Van der waals Pi-alkyl
Latamoxef (DB04570)		−7.4	Tyr-A:606 Asn-A:609 Gly-A:604 Ile-A:797 His-A:798 Ser-A:661 His-A:711	4.52 4.04 3.32 5.24 4.87 5.27	Van der waals Conventional Hydrogen bond Carbon hydrogen bond Alkyl
Cyclothiazide (DB00606)		−7.3	Phe-A:485 Val-A:603 Gly-A:604 Tyr-A:606 Asn-A:492 Gln-A:602 Thr-A:605	6.04 3.80 3.69 3.26 5.00 5.29 4.42	Van der waals Conventional Hydrogen bond Unfavourable Donor-Donor Alkyl Pi-Alkyl

**FIGURE 9:**
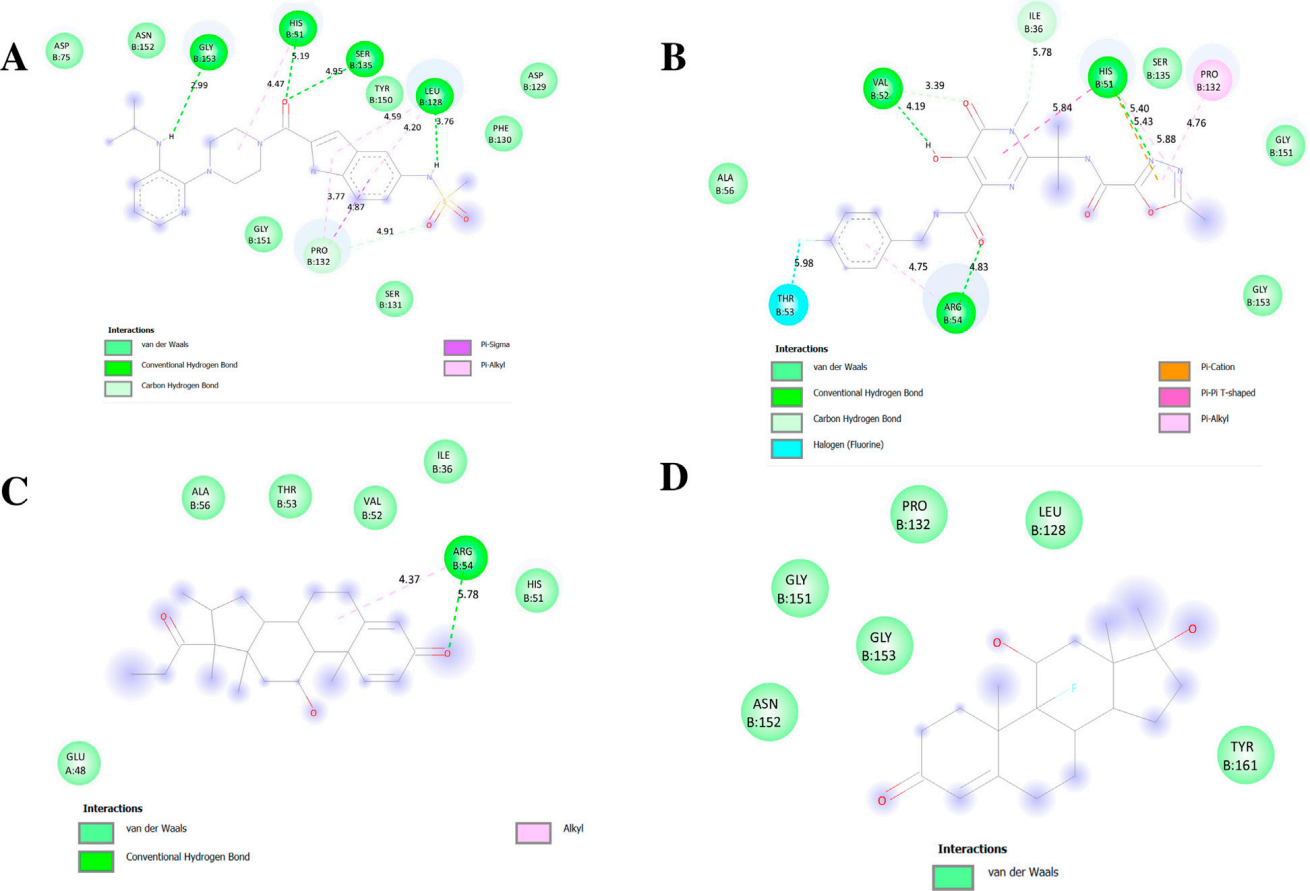
Docking poses of selected drugs **(A)** Delavirdine; **(B)** Raltegravir; **(C)** Rimexolone and **(D)** Fluoxymesterone with DENV NS2BNS3 protease represented as 2-D line models.

The NS2B-NS3 protease contains a catalytic triad (His51, Asp75, Ser135) in its active site (Aguilera-Pesantes et al., 2017; Lin et al., 2024; Norshidah et al., 2023). In our molecular docking analysis, Delavirdine and Raltegravir exhibited strong binding within the active site, suggesting their potential as NS2B-NS3 protease inhibitors. Delavirdine forms hydrogen bonds with His51 and Ser135, potentially interfering with catalytic activity, while Asp75 stabilizes His51. Additional interactions with Tyr150, Leu128, and Pro132 further enhance ligand stability within the active site (Figure 9A). Raltegravir engages His51 through Pi-Pi stacking and Pi-alkyl interactions, which may disrupt Ser135 activation. A hydrogen bond with Ser135 could impair its nucleophilic function, affecting protease activity. Additional hydrogen bonds with Thr53 and Arg54 reinforce Raltegravir binding (Figure 9B).

**FIGURE 10:**
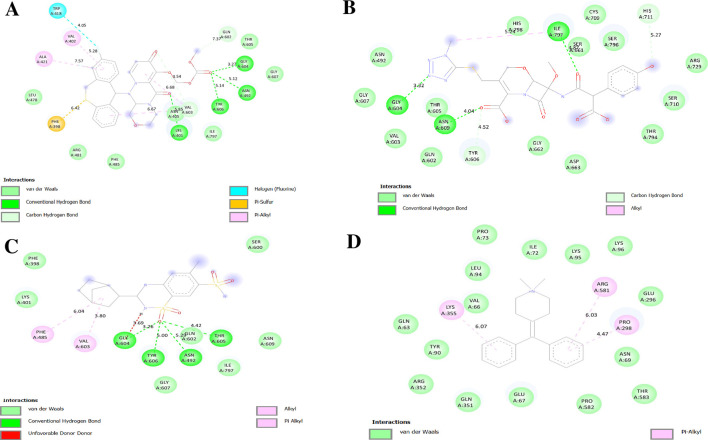
Docking poses of selected drugs **(A)** Baloxavir marboxil; **(B)** Latamoxef; **(C)** Cyclothiazide and **(D)** Diphemanil with DENV NS5 polymerase represented as 2-D line models.

The NS5 polymerase binding pocket comprises conserved residues (Ser56, Gly81, Cys82, Arg84, Gly85, Thr104, Lys105, His110, Asp131, Val132, Asp146, and Gly148) and a hydrophobic pocket containing Trp302, Phe354, Val358, Val577, Val579, Val603, Gly599, Ala406, Ala407, Asn492, Glu507, Tyr606, and Ile797 (Mukhtar et al., 2023; Valencia et al., 2021). Among the tested drugs, Baloxavir marboxil, Latamoxef, and Cyclothiazide exhibited strong binding, suggesting their potential as NS5 polymerase inhibitors. Baloxavir marboxil binds tightly to NS5 polymerase through hydrogen bonds with Asn492, Tyr606, Gly604, Thr605, Gln602, Gly607, and Val603, while hydrophobic interactions with Trp418, Val402, Ala421, and Phe398 enhance stability. The presence of conserved residues suggests high-affinity binding, potentially inhibiting NS5 activity (Figure 10A). Latamoxef forms hydrogen bonds with Asn492, Gly604, Thr605, Asn609, Tyr606, Ile797, His711, and Ser661, securing ligand stability. Additional interactions with Gly607 and Gln602 provide further stabilization, while Val603 and Ile797 contribute to hydrophobic anchoring, suggesting Latamoxef’s potential as an NS5 polymerase inhibitor (Figure 10B). Cyclothiazide interacts with NS5 polymerase via hydrogen bonds with Asn492, Tyr606, Gly604, Thr605, Gln602, and Gly607, stabilizing its binding. Pi-alkyl interactions with Val603 and Phe485 enhance ligand stability, while the involvement of Ile797 suggests strong anchoring within the active site, reinforcing Cyclothiazide’s inhibitory potential (Figure 10C).

**FIGURE 11:**
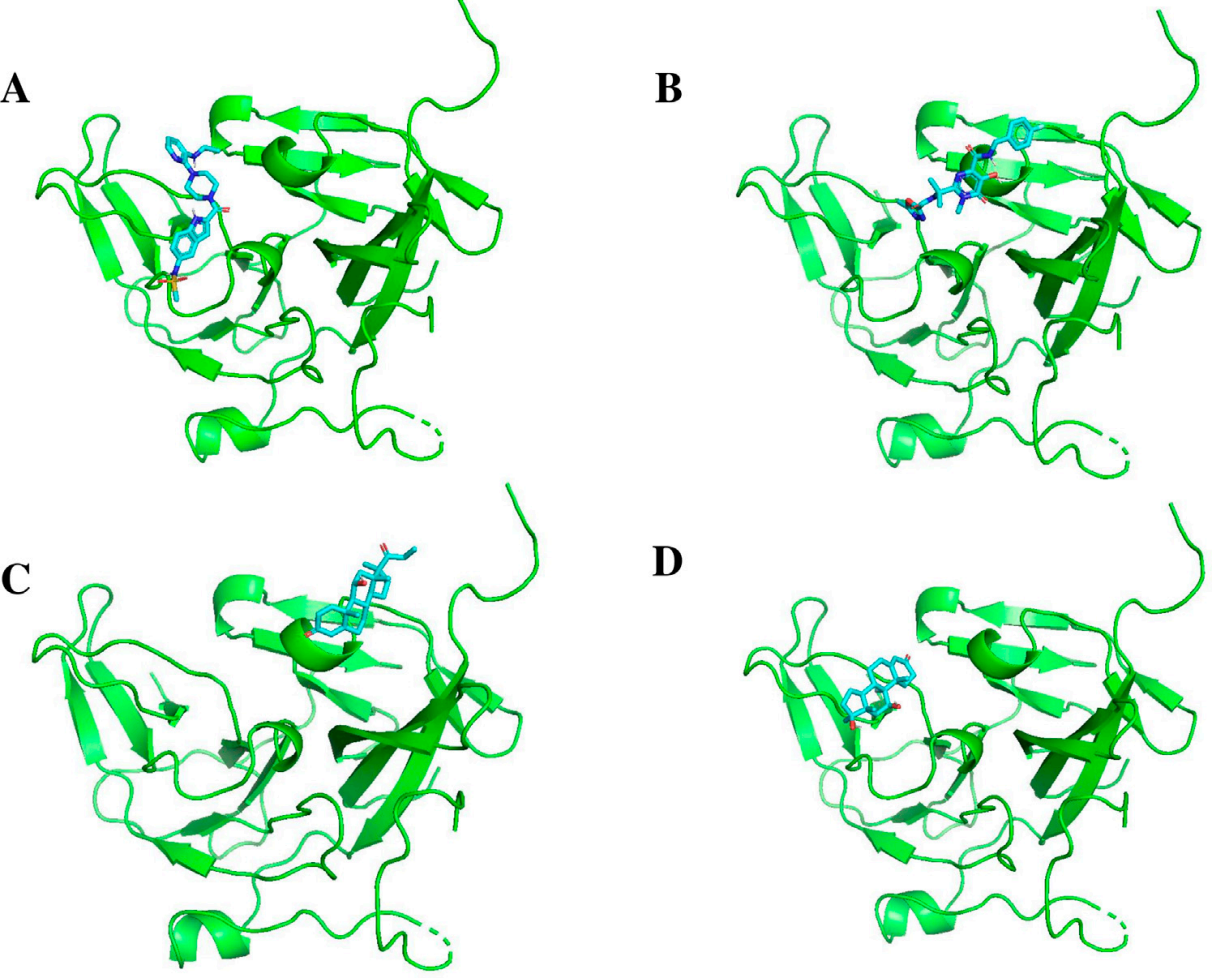
Docking poses of selected drugs **(A)** Delavirdine; **(B)** Raltegravir; **(C)** Rimexolone and **(D)** Fluoxymesterone with DENV NS2BNS3 protease in the form of ribbon structures.

**FIGURE 12:**
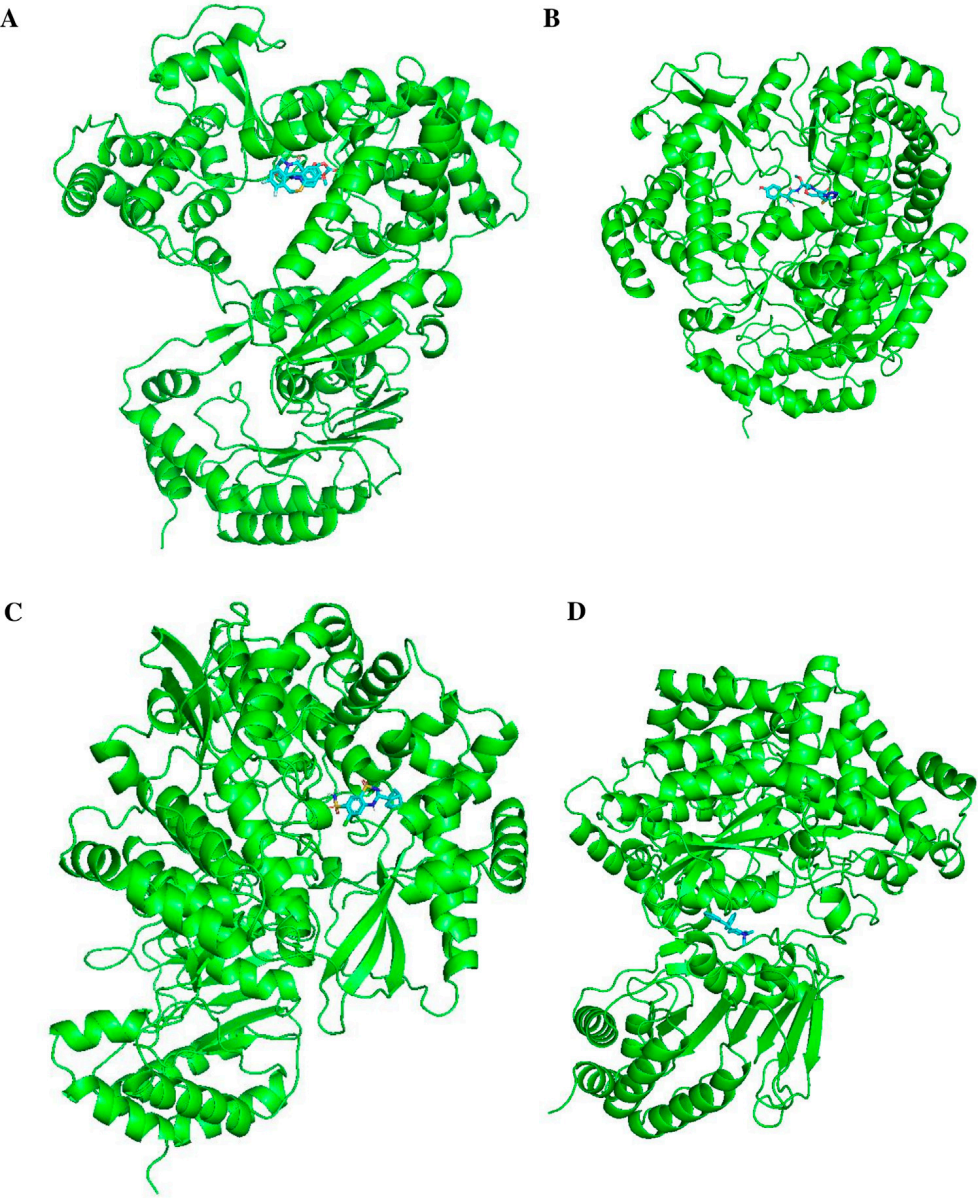
Docking poses of selected drugs **(A)** Baloxavir marboxil; **(B)** Latamoxef; **(C)** Cyclothiazide and **(D)** Diphemanil with DENV NS5 polymerase in the form of ribbon structures.

Figures 11, 12 display the ribbon structures of NS3 and NS5 proteins bound with their respective ligands, while Figures 9, 10 illustrate their molecular interactions in two-dimensional line models.

## Discussion

Dengue remains a major global health challenge due to the lack of effective antivirals and vaccine development difficulties (Norshidah et al., 2021). The genetic diversity among DENV serotypes (DENV-1–4) ranges from 60% to 75% amino acid homology, while viruses within the same serotype share over 97% (Guzman and Harris, 2015). Effective antivirals must target all serotypes to prevent antibody-dependent enhancement (ADE). Vaccine development is hindered by limited knowledge of protective factors, inadequate animal models, and the need for a tetravalent vaccine (Wilder-Smith, 2020). Further, natural products and phytochemicals have also been explored as an alternative to synthetic drugs for managing dengue fever (Sagar et al., 2024; Saleh and Kamisah, 2020). However, developing new small molecule antivirals is crucial, and employing a computational approach in this process would significantly accelerate drug discovery research. To address this, we developed “i-DENV”, a QSAR-based machine-learning pipeline predicting repurposed drugs against NS3 and NS5 proteins.

In this study, we employed SVM, RF, ANN, kNN, XGBoost, and DNN algorithms to develop predictive models, which have been widely utilized in previous studies. For instance, Auto-Kla identifies lysine lactylation sites (Lai and Gao, 2023), and IML-TYLCVs assesses TYLCV symptom severity (Bupi et al., 2023). Similarly, MLTs have been applied in various antiviral prediction platforms, including AVPpred (Thakur et al., 2012), AVP-IC50Pred (Qureshi et al., 2015), SMEpred (Dar et al., 2016), HIVprotI (Qureshi et al., 2018), and Anti-nipah (Rajput et al., 2019), anti-Ebola (Rajput and Kumar, 2022), anti-corona (Rajput et al., 2021a), and anti-dengue (Gautam et al., 2024). But these servers primarily focus on predicting antiviral candidates against entire viruses, lacking target protein specificity. However, few research groups have also employed *in silico* methods like molecular docking, ligand interaction analysis, and QSAR-based models to identify repurposed drugs against DENV (Panchal et al., 2021) (Pathak et al., 2017) (Tahir ul Qamar et al., 2019). For instance, Chongjun et al. developed a classification model using 591 NS3-targeting compounds, generating nine molecular fingerprints. They implemented SVM, RF, and XGBoost models for evaluating accuracy and ROC_AUC (Valencia et al., 2021). Similarly, Kurniawan et al. created a classification model using 845 compounds, 2,603 molecular descriptors and applied ensemble RF, AdaBoost, and ERT models. Further, they evaluated performance using accuracy, AUC, MCC, etc. (Kurniawan et al., 2020). However, our study advances previous work by using a larger dataset and extending the analysis by including NS5 protein. Unlike classification approaches, we employed regression models evaluated by MAE, MSE, RMSE, *R* ^2^, and PCC. Our comprehensive validation includes applicability domain analysis, scatter plots, decoy validation, chemical clustering, and further support by molecular docking approach. The best SVM-SVR models were integrated on the web server to predict inhibitors specifically targeting DENV NS3 and NS5 proteins. Thus, “i-DENV” represents the first regression-based web server to identify potential drug candidates against the NS3 and NS5 proteins of DENV.

We have also predicted potential repurposed drug candidates from the approved DrugBank database using our best models for both NS3 and NS5 proteins. The top predicted drugs have also shown antiviral effects against other viruses. For example, in case of NS3, Micafungin and its derivatives are effective against SARS-CoV-2 (Nakajima et al., 2023), chikungunya (Ho et al., 2018), PRV (Hondo et al., 2024), and enterovirus 71 (Kim et al., 2016). Trametinib inhibits influenza A (Schräder et al., 2018), while Ergotamine blocks TMPRSS2 activity relevant to COVID-19 (Wang et al., 2022). Candesartan cilexetil, and Montelukast targets Zika (Loe et al., 2019) (Chen et al., 2020), SARS-CoV-2 (Copertino et al., 2021), and MERS-CoV (Gan et al., 2021). For NS5, Doxycycline inhibits COVID-19 (Gendrot et al., 2020), vesicular stomatitis virus (Wu et al., 2015), and JEV (Topno and Khan, 2018). Ribavirin is effective against HCV (Dixit and Perelson, 2006), PPRV (Zhang et al., 2023), and IBDV (Akram et al., 2023). Minocycline, combined with favipiravir, may treat COVID-19 (Itoh et al., 2022) and inhibits RSV (Bawage et al., 2019) and West Nile virus (Michaelis et al., 2007). Heparan sulfate mimetics are active against enterovirus 71 (Pourianfar et al., 2012), African swine fever virus (Garcfa-Villal and Gil-Fern, 1991), and HIV (Baba et al., 1988). Doxorubicin shows anti-chikungunya activity (Kasabe et al., 2023), while Posaconazole inhibits the SARS-CoV-2 helicase (Abidi et al., 2021).

Further, several top candidates for both targets have been previously reported in the literature through experimental or *in silico* analyses for DENV. For NS3, Micafungin (IC_50_–10.23 μM) and its analogs Caspofungin (IC_50_–20.78 μM) and Anidulafungin (IC_50_–3.24 μM) exhibit antiviral effects (Chen et al., 2021). Desmopressin was identified as a potential NS5 inhibitor via pharmacophore modeling (Kumar et al., 2022). AR-12, a celecoxib derivative, inhibits all DENV serotypes (Hassandarvish et al., 2017). Trametinib suppresses flaviviral replication (Valencia et al., 2021). Ergotamine and conivaptan exhibit high *in silico* binding affinity (Montes-Grajales et al., 2020). Candesartan cilexetil inhibits DENV-2 (IC_50_–1.602 μM) (Loe et al., 2019) and Cyclosporine disrupts NS5-Cyclophilin interaction, hindering replication (Qing et al., 2009). Montelukast inhibits NS2B-NS3 protease (IC_50_–25.65 mM) (Jiang et al., 2022). For NS5, doxycycline inhibits all DENV serotypes (IC_50_–52.3 μM at 37°C, 26.7 μM at 40°C), with greater efficacy against DENV2 and DENV4 (Rothan et al., 2014). Mycophenolic acid (IC_50_–0.4 mM) and ribavirin (IC_50_–50.9 mM) inhibit DENV2 replication (Takhampunya et al., 2006). Minocycline targets multiple DENV life cycle stages (Leela et al., 2016), while sulfated polysaccharides show varying antiviral activity (PPS < suramin < PI-88) (Lee et al., 2006). Doxorubicin (CC_50_–116.9 μM, EC_50_–6.573 μM) and posaconazole block DENV RNA replication (Punekar et al., 2022) (Meutiawati et al., 2018). Thus, the repurposed drug candidates predicted by our algorithm hold promise as antiviral agents, potentially accelerating DENV drug discovery efforts. Further, molecular docking validated the top predicted drugs’ interactions with NS3 and NS5 proteins. Delavirdine showed the lowest NS3 binding energy (−8.3 kcal/mol), interacting with catalytic residues (His51, Ser135), while Baloxavir marboxil had the lowest NS5 energy (−8.4 kcal/mol), forming stable hydrogen bonds and hydrophobic interactions. These results confirm the model’s accuracy in predicting DENV inhibitors.

The key feature of the current study is the development of the “i-DENV” web server, specifically designed to predict the antiviral activity of molecules against DENV. For algorithm development, we utilized an experimentally tested dataset of molecules targeting NS3 and NS5 proteins of DENV. Additionally, we also predict potential repurposed drug candidates that were also previously reported in the literature. “i-DENV” is the first regression-based web server designed to assess the efficacy of user-defined molecules targeting both NS3 and NS5 proteins.

The limitation of the current study is the relatively small dataset used for the NS5 protein; expanding the dataset could further enhance the model’s predictive performance. Another limitation is that the “i-DENV” algorithm is designed solely to predict the antiviral efficacy of a molecule in terms of its pIC_50_ value. In the future, this approach could be extended to multi-task learning (MTL) by incorporating additional properties beyond antiviral activity prediction. To ensure the reliable prediction of our model, experimental validation of a molecule is essential to confirm their efficacy against DENV.

## Conclusion

“i-DENV” algorithm was developed using QSAR properties of compounds and employing various MLTs (SVM, RF, kNN, ANN, XGBoost and DNN). We created 360 unique configurations (5 random states × 4 feature sets × 3 feature selection methods × 6 models per set) for each protein dataset (NS3 and NS5). For NS3, the SVM-SVR and ANN-SVR models demonstrated PCC values of 0.857 and 0.862 on the TT dataset, and 0.870 and 0.894 on the IV dataset, respectively. For NS5, SVM-SVR and ANN-SVR showed PCC values of 0.982 and 0.964 on the TT dataset, and 0.970 and 0.977 on the IV dataset, respectively. These model’s robustness was confirmed through various analyses like applicability domain, chemical clustering, decoy set, statistical tests, etc. We used the approved category of the DrugBank database to identify potential repurposed drugs. Further, the top candidates were validated through molecular docking that confirmed the reliability of the predictive models. While the findings provide promising insights, it is important to note that the study is entirely based on *in silico* methodologies. Therefore, further *in vitro* and *in vivo* experimental validation is essential to confirm the therapeutic efficacy of the predicted compounds. Overall, the freely accessible “i-DENV” web server serves as a valuable resource for identifying novel antiviral candidates targeting the non-structural proteins of the DENV.

## Data Availability

The datasets presented in this study can be found in online repositories. The names of the repository/repositories and accession number(s) can be found in the article/Supplementary Material.

## References

[B1] Abdullah Z. L. Chee H. Y. Yusof R. Mohd Fauzi F. (2023). Finding lead compounds for dengue antivirals from a collection of old drugs through *in silico* target prediction and subsequent *in vitro* validation. ACS Omega 8 (36), 32483–32497. 10.1021/acsomega.3c02607 37720780 PMC10500654

[B2] Abidi S. H. Almansour N. M. Amerzhanov D. Allemailem K. S. Rafaqat W. Ibrahim M. A. A. (2021). Repurposing potential of posaconazole and grazoprevir as inhibitors of SARS-CoV-2 helicase. Sci. Rep. 11 (1), 10290. 10.1038/s41598-021-89724-0 33986405 PMC8119689

[B3] Aguilera-Pesantes D. Robayo L. E. Méndez P. E. Mollocana D. Marrero-Ponce Y. Torres F. J. (2017). Discovering key residues of dengue virus NS2b-NS3-protease: new binding sites for antiviral inhibitors design. Biochem. Biophys. Res. Commun. 492 (4), 631–642. 10.1016/j.bbrc.2017.03.107 28343993

[B4] Akram T. Gul I. Parveez Zia M. Hassan A. Khatun A. Shah R. A. (2023). Ribavirin inhibits the replication of infectious bursal disease virus predominantly through depletion of cellular guanosine pool. Front. Vet. Sci. 10, 1192583. 10.3389/fvets.2023.1192583 37601760 PMC10433155

[B5] Baba M. Nakajima M. Schols D. Pauwels R. Balzarini J. De Clercq E. (1988). Pentosan polysulfate, a sulfated oligosaccharide, is a potent and selective anti-HIV agent *in vitro* . Antivir. Res. 9, 335–343. 10.1016/0166-3542(88)90035-6 2465736

[B6] Backman T. W. H. Cao Y. Girke T. (2011). ChemMine tools: an online service for analyzing and clustering small molecules. Nucleic Acids Res. 39 (Suppl. 2), W486–W491. 10.1093/nar/gkr320 21576229 PMC3125754

[B7] Balasubramanian A. Pilankatta R. Teramoto T. Sajith A. M. Nwulia E. Kulkarni A. (2019). Inhibition of dengue virus by curcuminoids. Antivir. Res. 162, 71–78. 10.1016/j.antiviral.2018.12.002 30529358 PMC6541004

[B8] Bawage S. S. Tiwari P. M. Pillai S. Dennis V. A. Singh S. R. (2019). Antibiotic minocycline prevents respiratory syncytial virus infection. Viruses 11 (8), 739. 10.3390/v11080739 31405261 PMC6723987

[B9] Behnam M. A. M. Nitsche C. Boldescu V. Klein C. D. (2016). The medicinal chemistry of Dengue virus. J. Med. Chem. Am. Chem. Soc. 59, 5622–5649. 10.1021/acs.jmedchem.5b01653 26771861

[B10] Bhatt S. Gething P. W. Brady O. J. Messina J. P. Farlow A. W. Moyes C. L. (2013). The global distribution and burden of dengue. Nature 496 (7446), 504–507. 10.1038/nature12060 23563266 PMC3651993

[B11] Biovia D. Berman H. Westbrook J. Feng Z. Gilliland G. Bhat T. (2016). Discovery studio visualizer, 17.2. San Diego: Dassault Systèmes.

[B12] Brady O. J. Gething P. W. Bhatt S. Messina J. P. Brownstein J. S. Hoen A. G. (2012). Refining the global spatial limits of dengue virus transmission by evidence-based consensus. PLoS Negl. Trop. Dis. 6 (8), e1760. 10.1371/journal.pntd.0001760 22880140 PMC3413714

[B13] Bupi N. Sangaraju V. K. Phan L. T. Lal A. Vo T. T. B. Ho P. T. (2023). An effective integrated machine learning framework for identifying severity of Tomato yellow leaf Curl virus and their experimental validation. Research 6, 0016. 10.34133/research.0016 36930763 PMC10013792

[B14] Cabarcas-Montalvo M. Maldonado-Rojas W. Montes-Grajales D. Bertel-Sevilla A. Wagner-Döbler I. Sztajer H. (2016). Discovery of antiviral molecules for dengue: *in silico* search and biological evaluation. Eur. J. Med. Chem. 110, 87–97. 10.1016/j.ejmech.2015.12.030 26807547

[B15] Cereto-Massagué A. Guasch L. Valls C. Mulero M. Pujadas G. Garcia-Vallvé S. (2012). DecoyFinder: an easy-to-use python GUI application for building target-specific decoy sets. Bioinformatics 28 (12), 1661–1662. 10.1093/bioinformatics/bts249 22539671

[B16] Chen Y. Li Y. Wang X. Zou P. (2020). Montelukast, an anti-asthmatic drug, inhibits zika virus infection by disrupting viral integrity. Front. Microbiol. 10, 10. 10.3389/fmicb.2019.03079 PMC7002393 32082265

[B17] Chen Y. C. Lu J. W. Yeh C. T. Lin T. Y. Liu F. C. Ho Y. J. (2021). Micafungin inhibits dengue virus infection through the disruption of virus binding, entry, and stability. Pharmaceuticals 14 (4), 338. 10.3390/ph14040338 33917182 PMC8067805

[B18] Copertino D. C. Duarte R. R. R. Powell T. R. de Mulder Rougvie M. Nixon D. F. (2021). Montelukast drug activity and potential against severe acute respiratory syndrome coronavirus 2 (SARS-CoV-2). J. Med. Virology 93, 187–189. 10.1002/jmv.26299 32658304 PMC7405283

[B19] Coulerie P. Nour M. Maciuk A. Eydoux C. Guillemot J. C. Lebouvier N. (2013). Structure-activity relationship study of biflavonoids on the dengue virus polymerase DENV-NS5 RdRp. Planta Med. 79 (14), 1313–1318. 10.1055/s-0033-1350672 23929244

[B20] Dar S. A. Gupta A. K. Thakur A. Kumar M. (2016). SMEpred workbench: a web server for predicting efficacy of chemicallymodified siRNAs. RNA Biol. 13 (11), 1144–1151. 10.1080/15476286.2016.1229733 27603513 PMC5100349

[B21] Dash P. K. Parida M. M. Saxena P. Abhyankar A. Singh C. P. Tewari K. N. (2006). Reemergence of dengue virus type-3 (subtype-III) in India: implications for increased incidence of DHF and DSS. Virol. J. 3. 10.1186/1743-422X-3-55 16824209 PMC1559593

[B22] Davies M. Nowotka M. Papadatos G. Dedman N. Gaulton A. Atkinson F. (2015). ChEMBL web services: streamlining access to drug discovery data and utilities. Nucleic Acids Res. 43 (W1), W612–W620. 10.1093/nar/gkv352 25883136 PMC4489243

[B23] de Almeida R. R. Paim B. de Oliveira S. A. Souza A. S. Gomes A. C. P. Escuissato D. L. (2017). Dengue hemorrhagic fever: a state-of-the-art review focused in pulmonary involvement. Lung 195, 389–395. 10.1007/s00408-017-0021-6 28612239 PMC7102422

[B24] Dixit N. M. Perelson A. S. (2006). The metabolism, pharmacokinetics and mechanisms of antiviral activity of ribavirin against hepatitis C virus. Cell. Mol. Life Sci. 63, 832–842. 10.1007/s00018-005-5455-y 16501888 PMC11136426

[B25] Dwivedi V. D. Arya A. Yadav P. Kumar R. Kumar V. Raghava G. P. S. (2021). DenvInD: dengue virus inhibitors database for clinical and molecular research. Brief. Bioinform 22 (3), bbaa098. 10.1093/bib/bbaa098 32510549

[B26] Eberhardt J. Santos-Martins D. Tillack A. F. Forli S. (2021). AutoDock Vina 1.2.0: new docking methods, expanded force field, and python bindings. J. Chem. Inf. Model 61 (8), 3891–3898. 10.1021/acs.jcim.1c00203 34278794 PMC10683950

[B27] Ebi K. L. Nealon J. (2016). Dengue in a changing climate. Environ. Res. 151, 115–123. 10.1016/j.envres.2016.07.026 27475051

[B28] Gan H. J. Harikishore A. Lee J. Jeon S. Rajan S. Chen M. W. (2021). Antiviral activity against middle east respiratory syndrome coronavirus by montelukast, an anti-asthma drug. Antivir. Res. 185. 10.1016/j.antiviral.2020.104996 PMC7726485 33309540

[B29] Garcfa-Villal D. Gil-Fern C. (1991). Antiviral activity of sulfated polysaccharides against African swine fever virus. Antivir. Res. 15, 139–148. 10.1016/0166-3542(91)90031-l 1713439

[B30] Gautam S. Thakur A. Rajput A. Kumar M. (2024). Anti-dengue: a machine learning-assisted prediction of small molecule antivirals against dengue virus and implications in drug repurposing. Viruses 16 (1), 45. 10.3390/v16010045 PMC10818795 38257744

[B31] Gendrot M. Andreani J. Jardot P. Hutter S. Delandre O. Boxberger M. (2020). *In vitro* antiviral activity of doxycycline against SARS-CoV-2. Molecules 25 (21), 5064. 10.3390/molecules25215064 33142770 PMC7663271

[B32] Gholami B. Norton I. Tannenbaum A. R. Agar N. Y. R. (2012). “Recursive feature elimination for brain tumor classification using desorption electrospray ionization mass spectrometry imaging,” in Proceedings of the Annual International Conference of the IEEE Engineering in Medicine and Biology Society, San Diego, CA, USA, 28 August 2012 - 01 September (IEEE), 5258–5261. 10.1109/EMBC.2012.6347180 PMC3649005 23367115

[B33] Goethals O. Kaptein S. J. F. Kesteleyn B. Bonfanti J. F. Van Wesenbeeck L. Bardiot D. (2023). Blocking NS3–NS4B interaction inhibits dengue virus in non-human primates. Nature 615 (7953), 678–686. 10.1038/s41586-023-05790-6 36922586 PMC10033419

[B34] Grisoni F. Ballabio D. Todeschini R. Consonni V. (2018). Molecular descriptors for structure–activity applications: a hands-on approach. Methods Mol. Biol. 1800, 3–53. 10.1007/978-1-4939-7899-1_1 29934886

[B35] Gupta G. Khan S. Guleria V. Almjally A. Alabduallah B. I. Siddiqui T. (2023). DDPM: a dengue disease prediction and diagnosis model using sentiment analysis and machine learning algorithms. Diagnostics 13 (6), 1093. 10.3390/diagnostics13061093 36980401 PMC10047105

[B36] Guzman M. G. Harris E. (2015). “Dengue,” in The lancet (London, UK: Lancet Publishing Group), 453–465.

[B37] Hassandarvish P. Oo A. Jokar A. Zukiwski A. Proniuk S. Bakar S. A. (2017). Exploring the *in vitro* potential of celecoxib derivative AR-12 as an effective antiviral compound against four dengue virus serotypes. J. Antimicrob. Chemother. 72 (9), 2438–2442. 10.1093/jac/dkx191 28666323

[B38] Hazra A. (2017). Using the confidence interval confidently. J. Thorac. Dis. 9 (10), 4125–4130. 10.21037/jtd.2017.09.14 29268424 PMC5723800

[B39] Ho Y. J. Liu F. C. Yeh C. T. Yang C. M. Lin C. C. Lin T. Y. (2018). Micafungin is a novel anti-viral agent of chikungunya virus through multiple mechanisms. Antivir. Res. 159, 134–142. 10.1016/j.antiviral.2018.10.005 30300716

[B40] Hondo E. Katta T. Sato A. Kadofusa N. Ishibashi T. Shimoda H. (2024). Antiviral effects of micafungin against pteropine orthoreovirus, an emerging zoonotic virus carried by bats. Virus Res. 339. 10.1016/j.virusres.2023.199248 PMC10665676 37858730

[B41] Indu P. Arunagirinathan N. Rameshkumar M. R. Sangeetha K. Divyadarshini A. Rajarajan S. (2021). Antiviral activity of astragaloside II, astragaloside III and astragaloside IV compounds against dengue virus: computational docking and *in vitro* studies. Microb. Pathog., 152. 10.1016/j.micpath.2020.104563 33098932

[B42] Irwin J. J. Tang K. G. Young J. Dandarchuluun C. Wong B. R. Khurelbaatar M. (2020). ZINC20 - a free ultralarge-scale chemical database for ligand discovery. J. Chem. Inf. Model 60 (12), 6065–6073. 10.1021/acs.jcim.0c00675 33118813 PMC8284596

[B43] Itoh K. Sakamaki I. Hirota T. Iwasaki H. (2022). Evaluation of minocycline combined with favipiravir therapy in coronavirus disease 2019 patients: a case-series study. J. Infect. Chemother. 28 (1), 124–127. 10.1016/j.jiac.2021.09.016 34627706 PMC8486618

[B44] Jarerattanachat V. Boonarkart C. Hannongbua S. Auewarakul P. Ardkhean R. (2023). *In silico* and *in vitro* studies of potential inhibitors against dengue viral protein NS5 methyl transferase from ginseng and notoginseng. J. Tradit. Complement. Med. 13 (1), 1–10. 10.1016/j.jtcme.2022.12.002 36685072 PMC9845645

[B45] Jiang H. Zhang Y. Wu Y. Cheng J. Feng S. Wang J. (2022). Identification of montelukast as flavivirus NS2B-NS3 protease inhibitor by inverse virtual screening and experimental validation. Biochem. Biophys. Res. Commun. 606, 87–93. 10.1016/j.bbrc.2022.03.064 35339757

[B46] Kaptein S. J. F. Goethals O. Kiemel D. Marchand A. Kesteleyn B. Bonfanti J. F. (2021). A pan-serotype dengue virus inhibitor targeting the NS3–NS4B interaction. Nature 598 (7881), 504–509. 10.1038/s41586-021-03990-6 34616043

[B47] Kar S. Roy K. Leszczynski J. (2018). Applicability domain: a step toward confident predictions and decidability for QSAR modeling. Methods Mol. Biol. 1800, 141–169. 10.1007/978-1-4939-7899-1_6 29934891

[B48] Kasabe B. Ahire G. Patil P. Punekar M. Davuluri K. S. Kakade M. (2023). Corrigendum: drug repurposing approach against chikungunya virus: an *in vitro* and *in silico* study. Front. Cell Infect. Microbiol. 13, 1226054. 10.3389/fcimb.2023.1226054 37424775 PMC10329110

[B49] Khan M. B. Yang Z. S. Lin C. Y. Hsu M. C. Urbina A. N. Assavalapsakul W. (2023). Dengue overview: an updated systemic review. J. Infect. Public Health 16, 1625–1642. 10.1016/j.jiph.2023.08.001 37595484

[B50] Khan R. A. Hossain R. Siyadatpanah A. Al-Khafaji K. Khalipha A. B. R. Dey D. (2021). Diterpenes/diterpenoids and their derivatives as potential bioactive leads against dengue virus: a computational and network pharmacology study. Molecules 26 (22), 6821. 10.3390/molecules26226821 34833913 PMC8623982

[B51] Khosrokhavar R. Ghasemi J. B. Shiri F. (2010). 2D quantitative Structure-property relationship study of mycotoxins by multiple linear regression and support vector machine. Int. J. Mol. Sci. 11 (9), 3052–3068. 10.3390/ijms11093052 20957079 PMC2956080

[B52] Kim C. Kang H. Kim D. E. Song J. H. Choi M. Kang M. (2016). Antiviral activity of micafungin against enterovirus 71. Virol. J. 13 (1), 99. 10.1186/s12985-016-0557-8 27296985 PMC4907259

[B53] Knox C. Wilson M. Klinger C. M. Franklin M. Oler E. Wilson A. (2024). DrugBank 6.0: the drugBank knowledgebase for 2024. Nucleic Acids Res. 52 (D1), D1265–D1275. 10.1093/nar/gkad976 37953279 PMC10767804

[B54] Kumar S. Bajrai L. H. Faizo A. A. Khateb A. M. Alkhaldy A. A. Rana R. (2022). Pharmacophore-model-based drug repurposing for the identification of the potential inhibitors targeting the allosteric site in dengue virus NS5 RNA-dependent RNA polymerase. Viruses 14 (8), 1827. 10.3390/v14081827 36016449 PMC9412353

[B55] Kurniawan I. Rosalinda M. Ikhsan N. (2020). Implementation of ensemble methods on QSAR study of NS3 inhibitor activity as anti-dengue agent. Sar. QSAR Environ. Res. 31 (6), 477–492. 10.1080/1062936X.2020.1773534 32546117

[B56] Kwon S. Bae H. Jo J. Yoon S. (2019). Comprehensive ensemble in QSAR prediction for drug discovery. BMC Bioinforma. 20 (1), 521. 10.1186/s12859-019-3135-4 PMC6815455 31655545

[B57] Lai F. L. Gao F. (2023). Auto-kla: a novel web server to discriminate lysine lactylation sites using automated machine learning. Brief. Bioinform 24 (2), bbad070. 10.1093/bib/bbad070 36869843

[B58] Lee E. Pavy M. Young N. Freeman C. Lobigs M. (2006). Antiviral effect of the heparan sulfate mimetic, PI-88, against dengue and encephalitic flaviviruses. Antivir. Res. 69 (1), 31–38. 10.1016/j.antiviral.2005.08.006 16309754

[B59] Leela S. L. Srisawat C. Sreekanth G. P. Noisakran S. Yenchitsomanus P. Limjindaporn T. (2016). Drug repurposing of minocycline against dengue virus infection. Biochem. Biophys. Res. Commun. 478 (1), 410–416. 10.1016/j.bbrc.2016.07.029 27396621

[B60] Lin X. Yang F. Zhou L. Yin P. Kong H. Xing W. (2012). A support vector machine-recursive feature elimination feature selection method based on artificial contrast variables and mutual information. J. Chromatogr. B Anal. Technol. Biomed. Life Sci. 910, 149–155. 10.1016/j.jchromb.2012.05.020 22682888

[B61] Lin Y. F. Lai H. C. Lin C. S. Hung P. Y. Kan J. Y. Chiu S. W. (2024). Discovery of potent dengue virus NS2B-NS3 protease inhibitors among glycyrrhizic acid conjugates with amino acids and dipeptides esters. Viruses 16 (12), 1926. 10.3390/v16121926 39772233 PMC11680386

[B62] Loe M. W. C. Lee R. C. H. Chu J. J. H. (2019). Antiviral activity of the FDA-approved drug candesartan cilexetil against zika virus infection. Antivir. Res. 172, 104637. 10.1016/j.antiviral.2019.104637 31669333

[B63] Lundberg S. M. Lee S. I. (2017). A unified approach to interpreting model predictions. Adv. Neural Inf. Process Syst. 2017 (2), 4766–4775. 10.48550/arXiv.1705.07874

[B64] Mendez D. Gaulton A. Bento A. P. Chambers J. De Veij M. Félix E. (2019). ChEMBL: towards direct deposition of bioassay data. Nucleic Acids Res. 47 (D1), D930–D940. 10.1093/nar/gky1075 30398643 PMC6323927

[B65] Messina J. P. Brady O. J. Golding N. Kraemer M. U. G. Wint G. R. W. Ray S. E. (2019). The current and future global distribution and population at risk of dengue. Nat. Microbiol. 4 (9), 1508–1515. 10.1038/s41564-019-0476-8 31182801 PMC6784886

[B66] Meutiawati F. Bezemer B. Strating JRPM Overheul G. J. Žusinaite E. van Kuppeveld F. J. M. (2018). Posaconazole inhibits dengue virus replication by targeting oxysterol-binding protein. Antivir. Res. 157, 68–79. 10.1016/j.antiviral.2018.06.017 29981375

[B67] Michaelis M. Kleinschmidt M. C. Doerr H. W. Cinatl J. (2007). Minocycline inhibits west nile virus replication and apoptosis in human neuronal cells. J. Antimicrob. Chemother. 60 (5), 981–986. 10.1093/jac/dkm307 17872917

[B68] Mirza S. B. Lee R. C. H. Chu J. J. H. Salmas R. E. Mavromoustakos T. Durdagi S. (2018). Discovery of selective dengue virus inhibitors using combination of molecular fingerprint-based virtual screening protocols, structure-based pharmacophore model development, molecular dynamics simulations and *in vitro* studies. J. Mol. Graph Model 79, 88–102. 10.1016/j.jmgm.2017.10.010 29156382

[B69] Montes-Grajales D. Puerta-Guardo H. Espinosa D. A. Harris E. Caicedo-Torres W. Olivero-Verbel J. (2020). *In silico* drug repurposing for the identification of potential candidate molecules against arboviruses infection. Antivir. Res., 173. 10.1016/j.antiviral.2019.104668 31786251

[B70] Mukhtar M. Khan H. A. Zaidi N. us S. S. (2023). Exploring the inhibitory potential of Nigella sativa against dengue virus NS2B/NS3 protease and NS5 polymerase using computational approaches. RSC Adv. 13 (27), 18306–18322. 10.1039/d3ra02613b 37333789 PMC10273825

[B71] Nakajima S. Ohashi H. Akazawa D. Torii S. Suzuki R. Fukuhara T. (2023). Antiviral activity of micafungin and its derivatives against SARS-CoV-2 RNA replication. Viruses 15 (2), 452. 10.3390/v15020452 36851666 PMC9958940

[B72] Nasar S. Rashid N. Iftikhar S. (2020). Dengue proteins with their role in pathogenesis, and strategies for developing an effective anti-dengue treatment: a review. J. Med. Virol. 92 (8), 941–955. 10.1002/jmv.25646 31784997

[B73] Natali E. N. Horst A. Meier P. Greiff V. Nuvolone M. Babrak L. M. (2024). Publisher correction: the dengue-specific immune response and antibody identification with machine learning. NPJ Vaccines 9 (1), 242. 10.1038/s41541-024-01027-3 39653698 PMC11628555

[B74] Nath S. Malakar P. Biswas B. Das S. Sabnam N. Nandi S. (2024). Exploring the targets of dengue virus and designs of potential inhibitors. Comb. Chem. High. Throughput Screen 27 (17), 2485–2524. 10.2174/0113862073247689231030153054 37962048

[B75] Norshidah H. Leow C. H. Ezleen K. E. Wahab H. A. Vignesh R. Rasul A. (2023). Assessing the potential of NS2B/NS3 protease inhibitors biomarker in curbing dengue virus infections: *in silico* vs. *in vitro* approach. Front. Cell. Infect. Microbiol. 13, 1061937. 10.3389/fcimb.2023.1061937 36864886 PMC9971573

[B76] Norshidah H. Vignesh R. Lai N. S. (2021). Updates on dengue vaccine and antiviral: where are we heading? Molecules 26, 6768. 10.3390/molecules26226768 34833860 PMC8620506

[B77] O’Boyle N. M. Banck M. James C. A. Morley C. Vandermeersch T. Hutchison G. R. (2011). Open babel: an open chemical toolbox. J. Cheminform 3 (10), 33. 10.1186/1758-2946-3-33 21982300 PMC3198950

[B78] Panchal R. Bapat S. Mukherjee S. Chowdhary A. (2021). *In silico* binding analysis of lutein and rosmarinic acid against envelope domain III protein of dengue virus. Indian J. Pharmacol. 53 (6), 471–479. 10.4103/ijp.IJP_576_19 34975135 PMC8764985

[B79] Pathak N. Lai M. L. Chen W. Y. Hsieh B. W. Yu G. Y. Yang J. M. (2017). Pharmacophore anchor models of flaviviral NS3 proteases lead to drug repurposing for DENV infection. BMC Bioinforma. 18, 548. 10.1186/s12859-017-1957-5 PMC5751397 29297305

[B80] Perkins R. Fang H. Tong W. Welsh W. J. (2003). Quantitative structure-activity relationship methods: perspectives on drug discovery and toxicology. Environ. Toxicol. Chem. 22, 1666–1679. 10.1897/01-171 12924569

[B81] Pourianfar H. R. Poh C. L. Fecondo J. Grollo L. (2012). *In vitro* evaluation of the antiviral activity of heparan sulfate mimetic compounds against enterovirus 71. Virus Res. 169 (1), 22–29. 10.1016/j.virusres.2012.06.025 22771616

[B82] Punekar M. Kasabe B. Patil P. Kakade M. B. Parashar D. Alagarasu K. (2022). A transcriptomics-based bioinformatics approach for identification and *in vitro* screening of FDA-Approved drugs for repurposing against dengue Virus-2. Viruses 14 (10), 2150. 10.3390/v14102150 36298705 PMC9609047

[B83] Qing M. Yang F. Zhang B. Zou G. Robida J. M. Yuan Z. (2009). Cyclosporine inhibits flavivirus replication through blocking the interaction between host cyclophilins and viral NS5 protein. Antimicrob. Agents Chemother. 53 (8), 3226–3235. 10.1128/AAC.00189-09 19451286 PMC2715601

[B84] Qureshi A. Kaur G. Kumar M. (2017). AVCpred: an integrated web server for prediction and design of antiviral compounds. Chem. Biol. Drug Des. 89 (1), 74–83. 10.1111/cbdd.12834 27490990 PMC7162012

[B85] Qureshi A. Rajput A. Kaur G. Kumar M. (2018). HIVprotI: an integrated web based platform for prediction and design of HIV proteins inhibitors. J. Cheminform 10 (1), 12. 10.1186/s13321-018-0266-y 29524011 PMC5845081

[B86] Qureshi A. Tandon H. Kumar M. (2015). AVP-IC50 pred: multiple machine learning techniques-based prediction of peptide antiviral activity in terms of half maximal inhibitory concentration (IC50). Biopolymers 104 (6), 753–763. 10.1002/bip.22703 26213387 PMC7161829

[B87] Rajput A. Kumar A. Kumar M. (2019). Computational identification of inhibitors using QSAR approach against nipah virus. Front. Pharmacol. 10 (FEB), 71. 10.3389/fphar.2019.00071 30809147 PMC6379726

[B88] Rajput A. Kumar A. Megha K. Thakur A. Kumar M. (2021b). DrugRepV: a compendium of repurposed drugs and chemicals targeting epidemic and pandemic viruses. Brief. Bioinform 22 (2), 1076–1084. 10.1093/bib/bbaa421 33480398 PMC7929368

[B89] Rajput A. Kumar M. (2018). Anti-flavi: a web platform to predict inhibitors of flaviviruses using QSAR and peptidomimetic approaches. Front. Microbiol. 9, 3121. 10.3389/fmicb.2018.03121 30619195 PMC6305493

[B90] Rajput A. Kumar M. (2022). Anti-ebola: an initiative to predict ebola virus inhibitors through machine learning. Mol. Divers 26 (3), 1635–1644. 10.1007/s11030-021-10291-7 34357513 PMC8343361

[B91] Rajput A. Thakur A. Mukhopadhyay A. Kamboj S. Rastogi A. Gautam S. (2021a). Prediction of repurposed drugs for coronaviruses using artificial intelligence and machine learning. Comput. Struct. Biotechnol. J. 19, 3133–3148. 10.1016/j.csbj.2021.05.037 34055238 PMC8141697

[B92] Rigsby R. E. Parker A. B. (2016). Using the PyMOL application to reinforce visual understanding of protein structure. Biochem. Mol. Biol. Educ. 44 (5), 433–437. 10.1002/bmb.20966 27241834

[B93] Rosner B. Glynn R. J. Lee M. L. T. (2006). The Wilcoxon signed rank test for paired comparisons of clustered data. Biometrics 62 (1), 185–192. 10.1111/j.1541-0420.2005.00389.x 16542245

[B94] Ross A. Willson V. L. (2017). Paired samples T-test. Basic and advanced statistical tests: writing results sections and creating tables and figures, 17–19.

[B95] Rothan H. A. Mohamed Z. Paydar M. Rahman N. A. Yusof R. (2014). Inhibitory effect of doxycycline against dengue virus replication *in vitro* . Arch. Virol. 159 (4), 711–718. 10.1007/s00705-013-1880-7 24142271

[B96] Sagar A. Agari M. Nandi S. (2024). “Natural drugs to combat dengue fever,” in Global trends in health, technology and management (Cham: Springer Nature Switzerland), 191–198.

[B97] Saleh M. S. M. Kamisah Y. (2020). Potential medicinal plants for the treatment of dengue fever and severe acute respiratory syndrome-coronavirus. Biomolecules 11 (1), 42. 10.3390/biom11010042 33396926 PMC7824034

[B98] Salleh H. M. Chong S. L. Othman R. Hazni H. Ahmad K. Yusof M. (2019). Dengue protease inhibition activity of selected Malaysian medicinal herbs. Trop. Biomed. 36, 357–366. 33597396

[B99] Schräder T. Dudek S. E. Schreiber A. Ehrhardt C. Planz O. Ludwig S. (2018). The clinically approved MEK inhibitor trametinib efficiently blocks influenza a virus propagation and cytokine expression. Antivir. Res. 157, 80–92. 10.1016/j.antiviral.2018.07.006 29990517

[B100] Shapiro S. S. Wilk A. M. B. (1965). An analysis of variance test for normality (complete samples). Biometrika 52, 591–611. 10.2307/2333709

[B101] Shimizu H. Saito A. Mikuni J. Nakayama E. E. Koyama H. Honma T. (2019). Discovery of a small molecule inhibitor targeting dengue virus NS5 RNA-dependent RNA polymerase. PLoS Negl. Trop. Dis. 13 (11), e0007894. 10.1371/journal.pntd.0007894 31738758 PMC6886872

[B102] Taghizadeh E. Heydarheydari S. Saberi A. JafarpoorNesheli S. Rezaeijo S. M. (2022). Breast cancer prediction with transcriptome profiling using feature selection and machine learning methods. BMC Bioinforma. 23 (1), 410. 10.1186/s12859-022-04965-8 PMC9526906 36183055

[B103] Tahir ul Qamar M. Maryam A. Muneer I. Xing F. Ashfaq U. A. Khan F. A. (2019). Computational screening of medicinal plant phytochemicals to discover potent pan-serotype inhibitors against dengue virus. Sci. Rep. 9 (1), 1433. 10.1038/s41598-018-38450-1 30723263 PMC6363786

[B104] Takhampunya R. Ubol S. Houng H. S. Cameron C. E. Padmanabhan R. (2006). Inhibition of dengue virus replication by mycophenolic acid and ribavirin. J. General Virology 87 (7), 1947–1952. 10.1099/vir.0.81655-0 16760396

[B105] Thakur N. Qureshi A. Kumar M. (2012). AVPpred: collection and prediction of highly effective antiviral peptides. Nucleic Acids Res. 40 (Web Server issue), W199–W204. 10.1093/nar/gks450 22638580 PMC3394244

[B106] Tomlinson S. M. Malmstrom R. D. Russo A. Mueller N. Pang Y. P. Watowich S. J. (2009). Structure-based discovery of dengue virus protease inhibitors. Antivir. Res. 82 (3), 110–114. 10.1016/j.antiviral.2009.02.190 19428601 PMC2680748

[B107] Topno R. Khan S. A. (2018). Efficacy of antibiotics (doxycycline and kanamycin) against Japanese encephalitis virus infection. Trop. Biomed. 35, 239–245. 33601796

[B108] Trott O. Olson A. J. (2010). AutoDock vina: improving the speed and accuracy of docking with a new scoring function, efficient optimization, and multithreading. J. Comput. Chem. 31 (2), 455–461. 10.1002/jcc.21334 19499576 PMC3041641

[B109] Valencia H. J. de Aguiar M. C. A. M. Costa M. A. Mendonça D. C. Reis E. V. Arias N. E. C. (2021). Evaluation of kinase inhibitors as potential therapeutics for flavivirus infections. Arch. Virol. 166 (5), 1433–1438. 10.1007/s00705-021-05021-1 33683474 PMC7938686

[B110] Van Der Maaten L. Hinton G. (2008). Visualizing data using t-SNE. J. Mach. Learn. Res. 9.

[B111] Waggoner J. J. Gresh L. Vargas M. J. Ballesteros G. Tellez Y. Soda K. J. (2016). Viremia and clinical presentation in nicaraguan patients infected with zika virus, chikungunya virus, and dengue virus. Clin. Infect. Dis. 63 (12), 1584–1590. 10.1093/cid/ciw589 27578819 PMC5146717

[B112] Wang S. Fang X. Wang Y. (2022). *In silico* screening of novel TMPRSS2 inhibitors for treatment of COVID-19. Molecules 27 (13), 4210. 10.3390/molecules27134210 35807455 PMC9268035

[B113] Wilder-Smith A. (2020). “Dengue vaccine development: status and future,”, 63. Springer, 40–44. 10.1007/s00103-019-03060-3 Bundesgesundheitsblatt - Gesundheitsforsch. - Gesundheitsschutz PMC7224137 31784763

[B114] Williamson D. F. Parker R. A. Kendrick J. S. (1989). The box plot: a simple visual method to interpret data. Ann. Intern. Med. 110, 916–921. 10.7326/0003-4819-110-11-916 2719423

[B115] Wu Z. C. Wang X. Wei J. C. Li B. B. Shao D. H. Li Y. M. (2015). Antiviral activity of doxycycline against vesicular stomatitis virus *in vitro* . FEMS Microbiol. Lett. 362 (22), fnv195. 10.1093/femsle/fnv195 26459887

[B116] Yap C. W. (2011). PaDEL-descriptor: an open source software to calculate molecular descriptors and fingerprints. J. Comput. Chem. 32 (7), 1466–1474. 10.1002/jcc.21707 21425294

[B117] Zhang W. Deng H. Liu Y. Chen S. Liu Y. Zhao Y. (2023). Ribavirin inhibits peste des petits ruminants virus proliferation *in vitro* . Vet. Med. (Praha) 68 (12), 464–476. 10.17221/56/2023-VETMED 38303996 PMC10828777

