# Supplementary material for: i-DENV: development of QSAR based regression models for predicting inhibitors targeting non-structural (NS) proteins of dengue virus
Source: Front Pharmacol. 2025 Jun 26;16:1605722. doi: 10.3389/fphar.2025.1605722 (PMC12241036; doi:10.3389/fphar.2025.1605722)
Supplement: Supplementary file 2 [file DataSheet1.docx]

**Supplementary Figures**


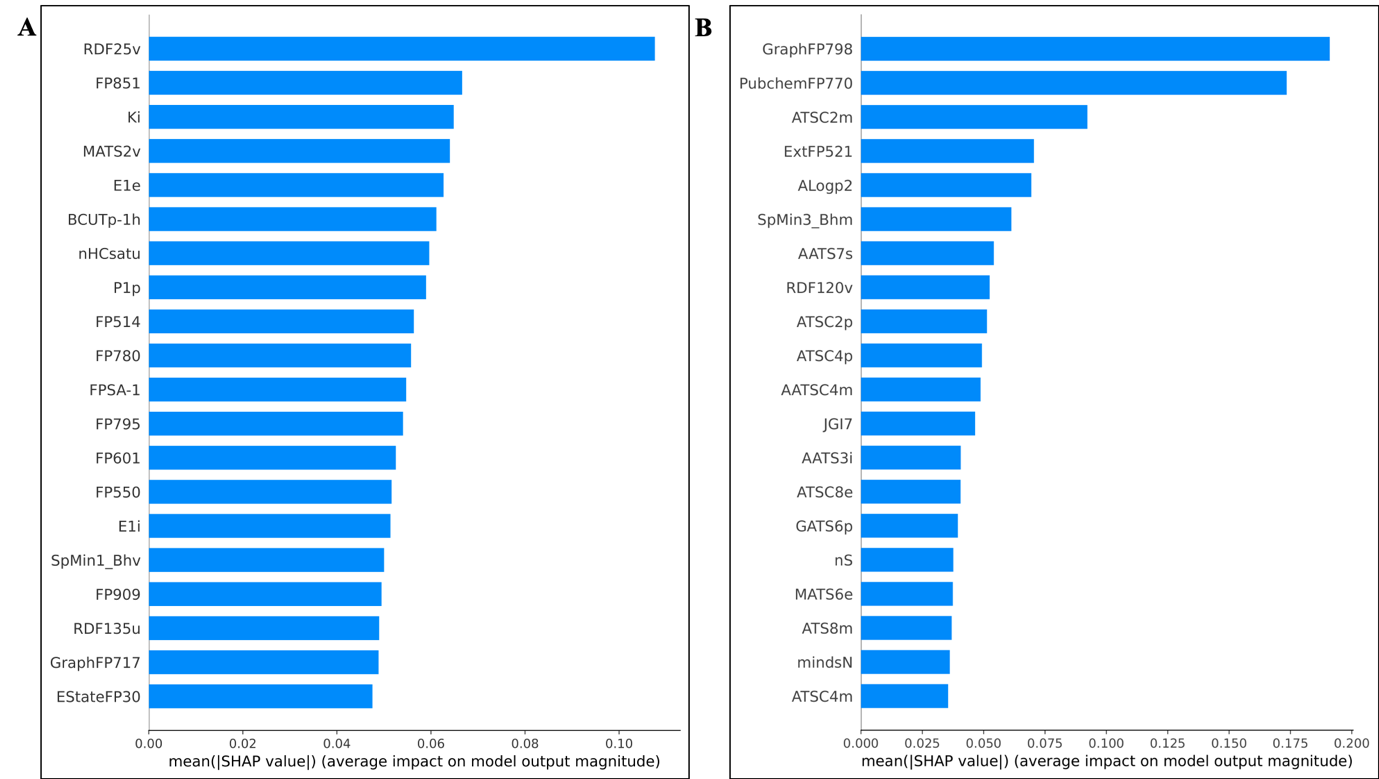


**Supplementary Figure 1** - Feature Importance Analysis Using SHAP Values in the Best Predictive SVM Model - (A) Mean SHAP Value Plot for NS3 Protein, (B) Mean SHAP Value Plot for NS5 Protein


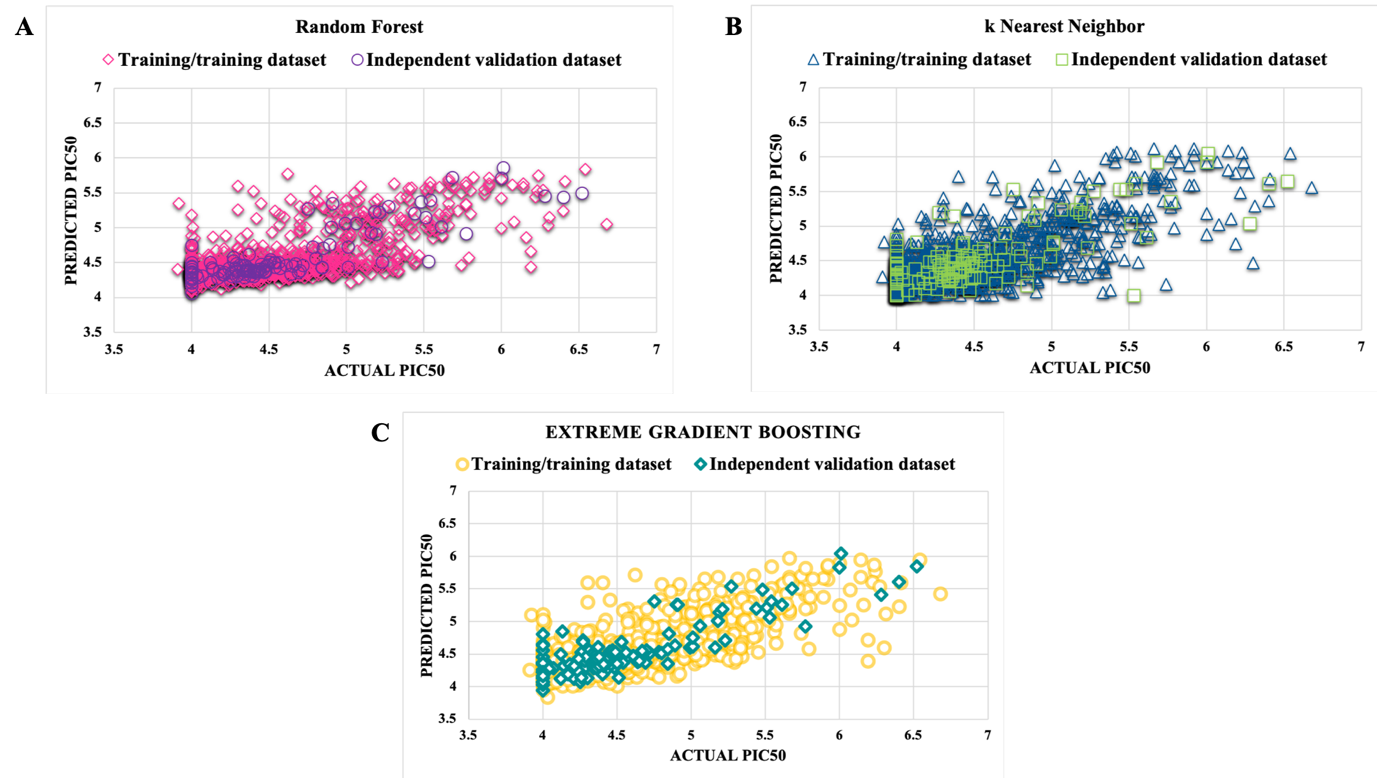


**Supplementary Figure 2** - Reliability of NS3-based predictive models, including (A) RF (B) kNN (C) XGBoost was evaluated by generating scatter plots that compare the actual pIC50 values of molecules with their corresponding predicted values.


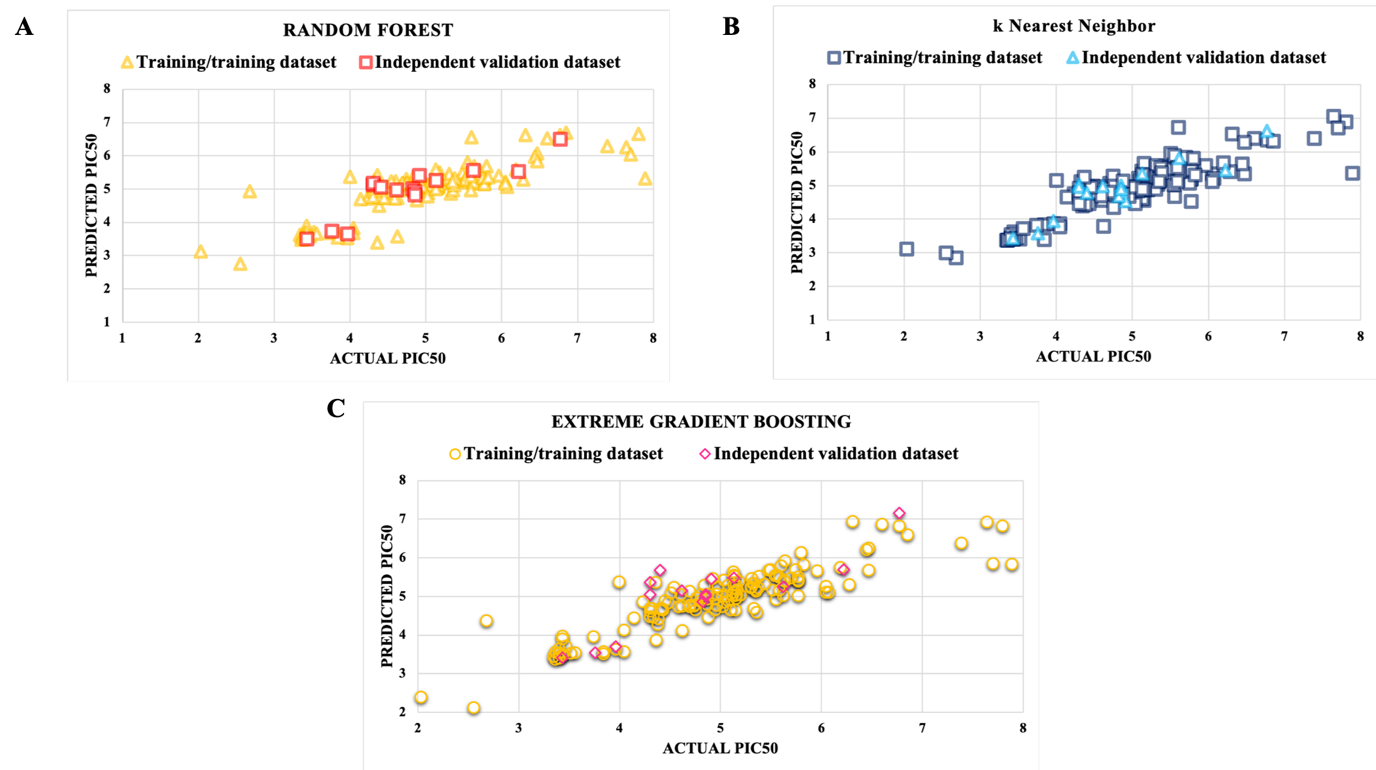


**Supplementary Figure 3** - Reliability of NS5-based predictive models, including (A) RF (B) kNN (C) XGBoost was evaluated by generating scatter plots that compare the actual pIC50 values of molecules with their corresponding predicted values.


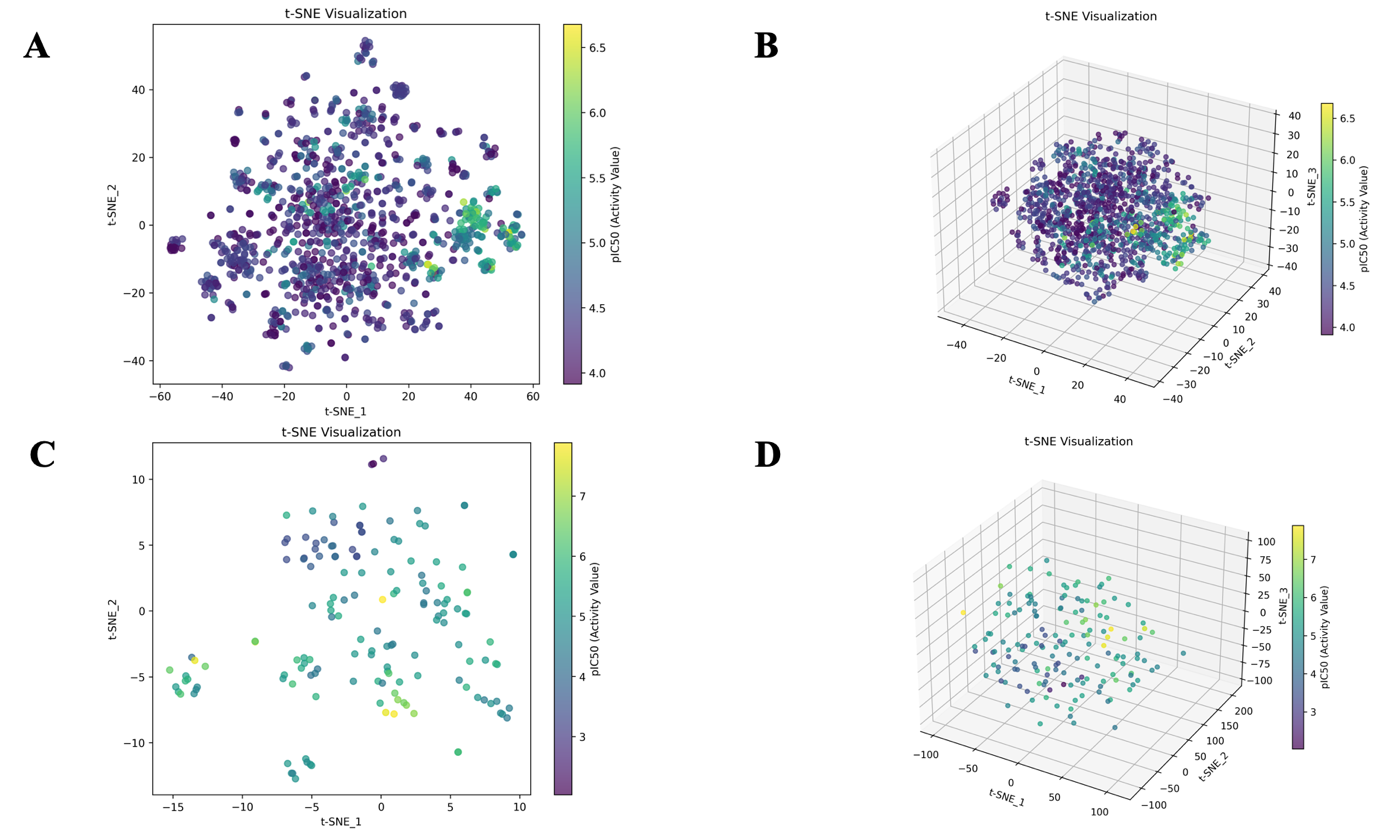


**Supplementary Figure 4** - Chemical Diversity Analysis Using t-SNE: (A) 2D t-SNE Plot for NS3, (B) 3D t-SNE Plot for NS3, (C) 2D t-SNE Plot for NS5, and (D) 3D t-SNE Plot for NS5.
